# The Rab7 effector PLEKHM1 binds Arl8b to promote cargo traffic to lysosomes

**DOI:** 10.1083/jcb.201607085

**Published:** 2017-04-03

**Authors:** Rituraj Marwaha, Subhash B. Arya, Divya Jagga, Harmeet Kaur, Amit Tuli, Mahak Sharma

**Affiliations:** 1 Department of Biological Sciences, Indian Institute of Science Education and Research, Mohali, Punjab 140306, India; 2 Division of Cell Biology and Immunology, Council of Scientific and Industrial Research, Institute of Microbial Technology, Chandigarh 160036, India

## Abstract

Endocytic, autophagic, and phagocytic vesicles move on microtubule tracks to fuse with lysosomes. Small GTPases, such as Rab7 and Arl8b, recruit their downstream effectors to mediate this transport and fusion. However, the potential cross talk between these two GTPases is unclear. Here, we show that the Rab7 effector PLEKHM1 simultaneously binds Rab7 and Arl8b, bringing about clustering and fusion of late endosomes and lysosomes. We show that the N-terminal RUN domain of PLEKHM1 is necessary and sufficient for interaction with Arl8b and its subsequent localization to lysosomes. Notably, we also demonstrate that Arl8b mediates recruitment of HOPS complex to PLEKHM1-positive vesicle contact sites. Consequently, Arl8b binding to PLEKHM1 is required for its function in delivery and, therefore, degradation of endocytic and autophagic cargo in lysosomes. Finally, we also show that PLEKHM1 competes with SKIP for Arl8b binding, which dictates lysosome positioning. These findings suggest that Arl8b, along with its effectors, orchestrates lysosomal transport and fusion.

## Abstract (precis)

Rab7 and Arl8b mediate vesicle transport and fusion with lysosomes. Marwaha et al. show that the Rab7 effector PLEKHM1 competes with PLEKHM2/SKIP for binding to Arl8b and that Arl8 mediates recruitment of the HOPS complex to PLEKHM1-positive vesicles for fusion, suggesting that Arl8b and its effectors orchestrate lysosomal transport and fusion.

## Introduction

Late endosome (LE) and lysosome motility and their fusion with other compartments are regulated by action of two small GTPases, Rab7 and Arl8b, and their numerous effectors, including adaptors, tethering factors, and microtubule-based motor-binding proteins (Wang et al., 2011; Khatter et al., 2015b). As with other members of the Rab and Arf-like (Arl) family, Rab7 and Arl8 cycle between inactive (GDP-bound) cytosolic and active (GTP-bound) membrane-bound conformations, recruiting their effectors to lysosomes in their GTP-bound state to mediate downstream functions.

Rab7, the better characterized of the two small GTPases, is primarily enriched on the LE/lysosome pool present in the perinuclear region of the cell near the microtubule organizing center (Wang et al., 2011). Herein, Rab7 recruits its effectors, RILP and PLEKHM1, to promote dynein-driven retrograde transport of LEs/lysosome and their fusion with endocytic, phagocytic, and autophagic vesicles (Jordens et al., 2001; McEwan et al., 2015a,b). RILP and PLEKHM1 interact with and recruit the multisubunit tethering factor HOPS complex to Rab7-positive LE/autophagosome–lysosome contact sites (van der Kant et al., 2013; Lin et al., 2014; McEwan et al., 2015a; Wijdeven et al., 2016). HOPS complex facilitates tethering of LEs/autophagosomes to lysosomes and binds with SNARE proteins to mediate membrane fusion (Balderhaar and Ungermann, 2013; Jiang et al., 2014). ORP1L, another Rab7 effector, induces formation of ER–LE membrane contact sites that inhibit recruitment of the PLEKHM1–HOPS complex to Rab7 (Rocha et al., 2009; Wijdeven et al., 2016). Finally, the Rab7 effector FYCO1 plays an opposing role to RILP by recruiting the motor protein kinesin-1 to promote anterograde movement of LEs/lysosomes (Pankiv et al., 2010).

Unlike Rab7, Arl8b is enriched on the peripheral lysosomes, which are less acidic and have reduced density of Rab7-RILP proteins on their surface (Hofmann and Munro, 2006; Johnson et al., 2016). Arl8b mediates anterograde lysosomal motility by recruiting SKIP (also known as PLEKHM2), which in turn recruits the motor protein kinesin-1 on lysosomes (Rosa-Ferreira and Munro, 2011). Recent studies have established that Arl8b-mediated positioning of lysosomes and lysosome-related organelles is important for nutrient sensing, cell migration, cancer cell metastasis, natural killer cell–mediated cytotoxicity, antigen presentation, and the formation of tubular lysosomes in macrophages (Korolchuk et al., 2011; Mrakovic et al., 2012; Tuli et al., 2013; Schiefermeier et al., 2014; Michelet et al., 2015; Dykes et al., 2016; Pu et al., 2016). Arl8b also regulates cargo trafficking to lysosomes by directly binding to the HOPS subunit Vps41, resulting in functional assembly of the HOPS complex on lysosomal membranes (Garg et al., 2011; Khatter et al., 2015a).

**Figure 1.**
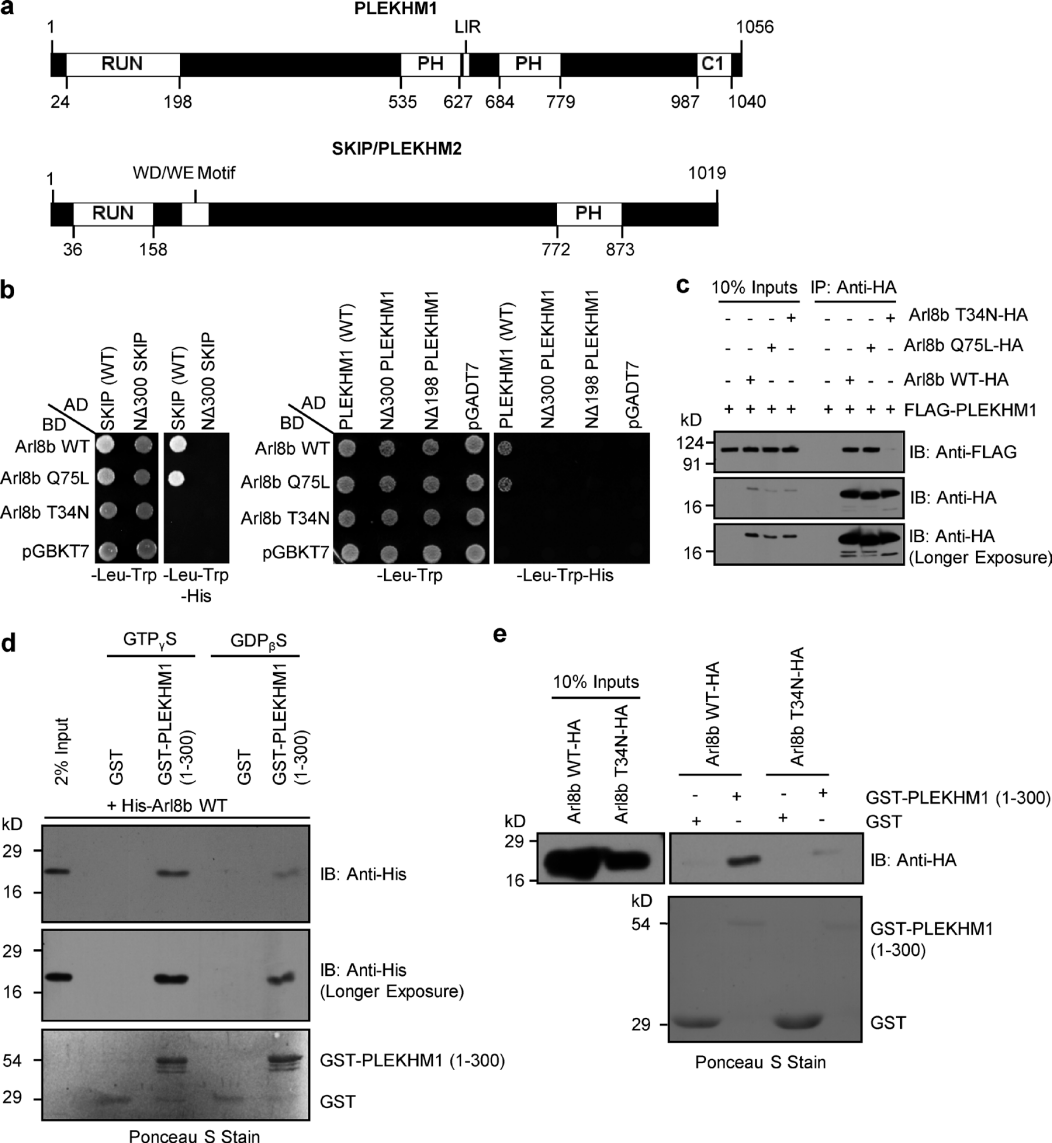
**PLEKHM1 directly binds to Arl8b via its N-terminal RUN domain–containing region.** (a) Domain architecture of PLEKHM1 and SKIP/PLEKHM2. (b) Yeast two-hybrid assay. Cotransformants were spotted on -Leu-Trp and -Leu-Trp-His media to confirm viability and interactions, respectively. (c) FLAG-PLEKHM1 was cotransfected with different forms of Arl8b-HA into HEK293T cells; lysates were immunoprecipitated (IP) with anti–HA antibody resin, and the precipitates were immunoblotted (IB) with the indicated antibodies. (d and e) GST and GST-PLEKHM1 (1–300) proteins were immobilized on glutathione (GSH) resin and incubated either with His-Arl8b in the presence of GTPγS or GDPβS or with HEK293T cell lysates expressing either Arl8b WT-HA or Arl8b T34N-HA. The precipitates were immunoblotted with anti–His (d) or anti–HA (e) antibodies. Ponceau S stain was done to visualize purified protein. LIR, LC3/GABARAP interaction; PH, pleckstrin homology; WD/WE, tryptophan-acidic.

Although Rab7 and Arl8b have an overlapping distribution and function, it is not known if they coordinate their activities. Previous studies suggest that dual or shared effectors represent a point of convergence of Rab, Arf, and Arl signals in membrane traffic (Burguete et al., 2008; Shi and Grant, 2013). In line with this, we noted that recently characterized Rab7 effector, PLEKHM1, shares ∼40% similarity over the length of its RUN domain with the known Arl8b effector SKIP. Importantly, it is the RUN domain that mediates SKIP binding to Arl8b. This prompted us to investigate whether PLEKHM1 can also interact with Arl8b using a similar binding interface as SKIP. PLEKHM1 was a plausible candidate for a dual Rab7/Arl8b effector as predicted from the distinct binding sites for the two GTPases; Arl8b binding mediated through its N-terminal RUN domain, whereas binding to Rab7 mediated via its C-terminal second PH domain and C1 zinc-finger domain (Fig. 1 a; Tabata et al., 2010; McEwan et al., 2015a). Here, we show that PLEKHM1 binds to Arl8b via its RUN domain to link the two GTPases. We identified conserved basic residues within the RUN domain required for binding to Arl8b. Using an Arl8b-binding–defective mutant of PLEKHM1 or cells lacking Arl8b, we show that (a) Arl8b is required for PLEKHM1 localization to lysosomes, but not LEs; (b) Arl8b mediates recruitment of the HOPS complex to Rab7/PLEKHM1-positive vesicle contact sites and consequently their clustering; and (c) Arl8b binding is crucial for PLEKHM1 to promote lysosomal degradation of endocytic and autophagic cargo. We also demonstrate that PLEKHM1 competes with SKIP for Arl8b binding and that the two effectors have opposing roles in regulating lysosome transport.

## Results

### PLEKHM1 directly binds to Arl8b via its N-terminal RUN domain–containing region

To investigate whether PLEKHM1 interacts with Arl8b via its RUN domain, we performed selective yeast two-hybrid assay with the full-length and a domain deletion mutant of PLEKHM1 lacking the N-terminal RUN domain-containing region (1–300 aa, NΔ300 PLEKHM1). We found that full-length PLEKHM1 interacted with the wild-type (WT) and Q75L (constitutively GTP-bound) forms of Arl8b, but not with the T34N (constitutively GDP-bound) form, indicating that PLEKHM1 interacts with Arl8b in its GTP-bound state (Fig. 1 b). No growth was observed between Arl8b and NΔ300 or a NΔ198 PLEKHM1 mutant (lacking only the RUN domain), demonstrating that interaction of PLEKHM1 with Arl8b was dependent on the presence of its RUN domain (Fig. 1 b). In the assay, WT and NΔ300 mutant of SKIP were used as controls to confirm the previously reported interaction of Arl8b with SKIP (Rosa-Ferreira and Munro, 2011; Fig. 1 b). We corroborated these findings in cells using coimmunoprecipitation experiments, where PLEKHM1 showed binding to WT and Q75L forms, but not the T34N form of Arl8b (Fig. 1 c). To further clarify that it is a direct interaction, GST and GST-tagged PLEKHM1 (1–300; first 300 aa) proteins were coincubated with His-tagged Arl8b in the presence of nonhydrolyzable GTP or GDP analogues, as well as with Arl8b-WT and T34N-expressing cell lysates. GST-PLEKHM1 (1–300) displayed a strong binding preference toward Arl8b in the presence of GTP, but not GDP (Fig. 1, d and e). We found similar binding of purified Arl8b to GST-PLEKHM1 (1–198; first 198 aa; Fig. S1 a). Because we consistently observed degradation of GST-PLEKHM1 (1–198) during its purification (Fig. S1 a, Ponceau S stain), we used PLEKHM1 (1–300) in our subsequent binding assays.

Arl8 family has two paralogs in higher vertebrates, Arl8a and Arl8b, both of which are 91% identical at the protein level, localized at the lysosomes, and have ubiquitous tissue expression (Khatter et al., 2015b). A previous study showed that both paralogs bound to SKIP through its RUN domain (Rosa-Ferreira and Munro, 2011). Surprisingly, we found a significantly weaker interaction of PLEKHM1 with Arl8a as compared with Arl8b, whereas similar binding to SKIP was observed for both paralogs (Fig. S1, b–e). These results suggest that the nonconserved residues between the Arl8 paralogs may play a role in determining the strength of effector binding. Together, these results demonstrate that the N-terminal RUN domain–containing region of PLEKHM1 is both necessary and sufficient for interaction with Arl8b.

### The RUN domain of PLEKHM1 is required for localization to Arl8b- and LAMP1-positive, but not Rab7-positive, endosomes

**Figure 2.**
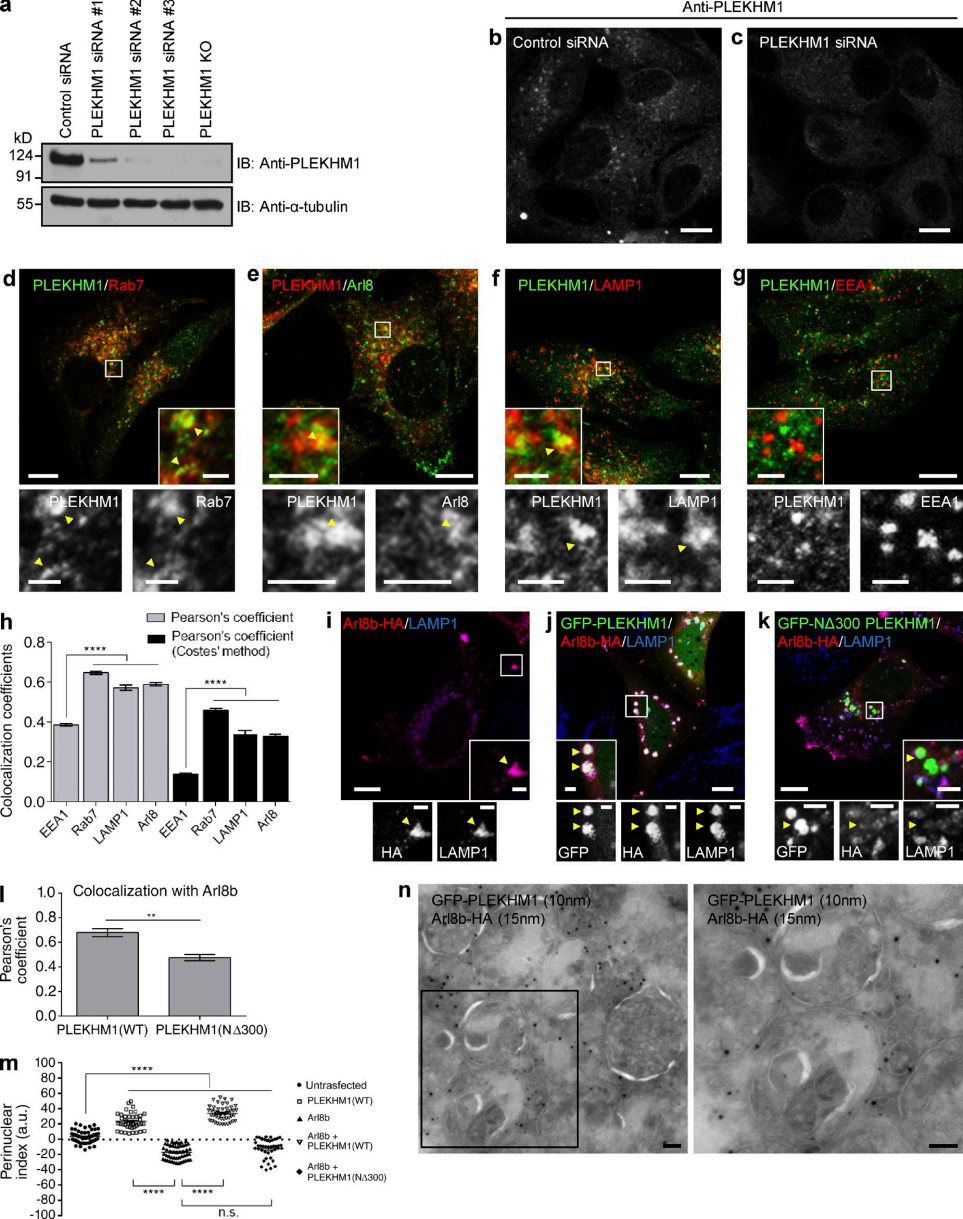
**PLEKHM1 colocalizes with Rab7 and Arl8b and promotes perinuclear clustering of lysosomes.** (a) Lysates from indicated siRNA treatments and from PLEKHM1 KO-HeLa cells were immunoblotted (IB) with anti-PLEKHM1 antibody for assessing the knockdown efficiency and α-tubulin as the loading control. (b and c) Immunofluorescence depicting the specificity of PLEKHM1 antibody in HeLa cells treated with control- and PLEKHM1-siRNA. (d–h) Representative confocal micrographs of HeLa cells showing endogenous staining of PLEKHM1 with different endocytic markers, and the Pearson’s correlation coefficient (PC) for PLEKHM1 is quantified (*n* = 3; 25–30 cells analyzed per experiment). (i–k) Representative confocal micrographs of HeLa cells transfected with Arl8b-HA alone or cotransfected with GFP-PLEKHM1 or -NΔ300 PLEKHM1, respectively, and stained for LAMP1. (l) Colocalization of WT and NΔ300 PLEKHM1 with Arl8b was assessed by measuring the PC (*n* = 3; 75 cells analyzed per experiment). (m) Quantification of perinuclear index of LAMP1^+^ compartments in HeLa cells transfected with indicated plasmids (*n* = 3; 15–18 cells analyzed per experiment). (n) Representative immunogold EM image of HeLa cells cotransfected with GFP-PLEKHM1 and Arl8b-HA and labeled with 10- and 15-nm gold particles, respectively. Boxed region is magnified on the right (Bar, 100 nm). Arrowheads mark colocalized pixels. Data represent mean ± SEM (n.s., not significant; **, P < 0.01; ****, P < 0.0001; Student’s *t* test). Bars: (main) 10 µm; (insets) 2 µm.

We next assessed the significance of Arl8b binding in PLEKHM1 localization and function. To visualize endogenous PLEKHM1 staining, we first verified the specificity of anti–PLEKHM1 antibody by confirming loss of signal intensity upon PLEKHM1-siRNA treatment or in PLEKHM1-knockout (KO) cells (Fig. 2, a–c). Although the signal-to-noise ratio was poor with this antibody, we were able to detect specific punctae that were absent in PLEKHM1-depleted cells (Fig. 2 c). As anticipated, several PLEKHM1-positive endosomes were colocalized with Rab7 (Fig. 2 d). Partial colocalization was also observed with LAMP1 and Arl8b. In comparison, we did not observe PLEKHM1 colocalization with EEA1, a marker for early endosomes (Fig. 2, e–h; and Fig. S1 n). Supporting its direct binding to Rab7 and Arl8b, endogenous PLEKHM1 was recruited to Rab7/Arl8b-labeled punctae in cells transfected with either of the two GTPases, whereas cytosolic staining was observed in cells transfected with dominant-negative Rab7 mutant (Rab7 T22N; Fig. S1, f–h). The aforementioned results further corroborated that the endosomal staining with anti–PLEKHM1 antibody were specific. We next assessed the significance of the RUN domain of PLEKHM1 in regulating its colocalization with Arl8b. In accordance with our observations that the RUN domain of PLEKHM1 was required for binding to Arl8b, colocalization of Arl8b and LAMP1 with NΔ300 PLEKHM1 was significantly reduced as compared with WT (Fig. 2, j, k, and l [quantification]). In contrast, NΔ300 PLEKHM1 continued to localize to Rab7-positive endosomes (Fig. 2 k and Fig. S1 m), suggesting that the RUN domain of PLEKHM1 is required for its association with Arl8b/LAMP1-positive endolysosomes/lysosomes, but not with Rab7-positive LEs. Our data indicate that Arl8b does not mediate membrane recruitment of PLEKHM1; rather, this role has been attributed to Rab7 (Tabata et al., 2010). Accordingly, PLEKHM1 continued to be endosomal in cells expressing Arl8b T34N, whereas in cells transfected with Rab7 T22N, PLEKHM1 was cytosolic and failed to colocalize with Arl8b (Fig. S1, g, i, and j). Accordingly, domain deletion mutants of PLEKHM1 known to be defective in binding Rab7 (McEwan et al., 2015a) were cytosolic (Fig. S1, k and l).

### Arl8b binding is required for PLEKHM1 to mediate clustering of LEs and Lysosomes

We observed that cotransfection of PLEKHM1 and Arl8b led to dramatic perinuclear clustering of LAMP1-positive compartments, whereas transfection of Arl8b alone promoted lysosome positioning at the cell periphery (Fig. 2, i, j, and m). This effect was restricted only to the late endocytic compartments, as the subcellular distribution of organelles, including early endosomes or Golgi, was not altered (Fig. S2, a and b). Interestingly, transfection of NΔ300 PLEKHM1 and Arl8b did not induce perinuclear clustering of LAMP1-positive endosomes. Rather, lysosome positioning to cell periphery was observed in these cells (Fig. 2, k and m). Using structured illumination microscopy and cryo–immunogold EM, we observed that Arl8b and PLEKHM1 were present on the limiting membranes of these enlarged and tightly clustered endolysosomal compartments along with LAMP1 (Fig. 2 n; and Fig. S2, c and e). Live-cell imaging experiments (described later in the text) showed that Rab7 was also present on these clustered endosomes along with Arl8b (Video 2). Both PLEKHM1 and Arl8b were enriched on the vertices of these docked endolysosomes (Fig. S2 c, arrowheads). In contrast, NΔ300 PLEKHM1 was present on endosomes distinct from Arl8b and LAMP1 (Fig. S2 d), which were likely to be Rab7-positive LEs (as depicted in Fig. S1 m).

**Figure 3.**
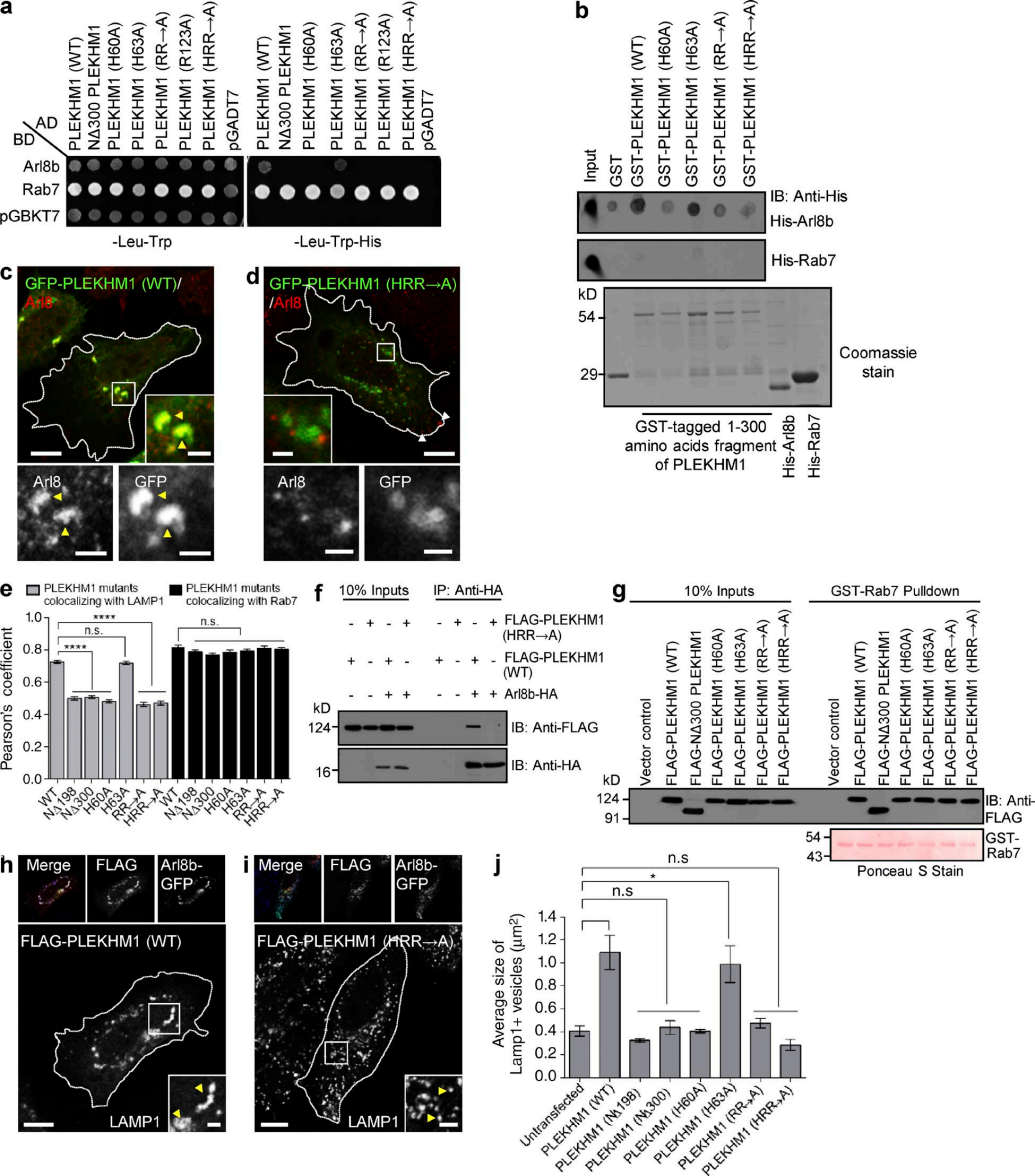
**Conserved basic residues within the RUN domain of PLEKHM1 are important for its interaction with Arl8b.** (a) Yeast two-hybrid assay. Cotransformants were spotted on -Leu-Trp and -Leu-Trp-His media to confirm viability and interactions, respectively. AD, GAL4 activation domain; BD, GAL4-DNA binding domain. (b) Dot-blot assay: GST alone or GST-PLEKHM1 (1–300) or indicated point mutants were spotted on nitrocellulose membrane and incubated with His-Arl8b or His-Rab7. The interaction was analyzed by immunoblotting (IB) with anti–His antibody. Proteins were visualized by Coomassie staining. (c and d) Representative confocal images showing HeLa cells transfected with GFP-PLEKHM1 or GFP-PLEKHM1 (HRR→A) and immunostained for Arl8. Yellow arrowheads mark colocalized pixels, and white arrowheads mark peripheral Arl8b^+^-lysosomes. (e) PC quantification of WT or mutant PLEKHM1 with LAMP1 and Rab7 (*n* = 3; 30 cells analyzed per experiment). (f) Arl8b-HA was cotransfected with FLAG-PLEKHM1 (WT) or HRR→A mutant in HEK293T cells. The lysates were immunoprecipitated (IP) using anti–HA antibody resin and immunoblotted using the indicated antibodies. (g) Immunoblot of a GST pulldown assay using HEK293T cell lysates expressing FLAG-PLEKHM1 (WT) or -Arl8b-binding–defective mutants of PLEKHM1 incubated with GST-Rab7 bound to GSH resin. GST-Rab7 protein was visualized by Ponceau S staining. (h and i) Representative confocal panels showing LAMP1 staining in HeLa cells cotransfected with Arl8b-GFP and FLAG-PLEKHM1 (WT) or HRR→A mutant. LAMP1 staining is shown in insets. (j) Mean size of LAMP1^+^ compartments in HeLa cells cotransfected with indicated PLEKHM1 plasmid and Arl8b-GFP (*n* = 3; 25 cells analyzed per experiment). Data represent mean ± SEM (n.s., not significant; *, P < 0.05; ****, P < 0.0001; Student’s *t* test). Bars: (main) 10 µm; (insets) 2 µm.

We next sought to identify the residues within the RUN domain of PLEKHM1 that might regulate Arl8b-binding. Sequence alignment of the conserved core of the RUN domain family members (organized in six blocks from A-F) has revealed polar amino acids within the RUN domain that may regulate interaction with the Ras superfamily of small GTPases (Callebaut et al., 2001). Sequence alignment of PLEKHM1 and SKIP RUN domain showed the conserved polar residues within these proteins (Fig. S2 f). To this end, we created single (H60A, H63A, and R123A), double (R117A/R119A; “RR→A”) or triple (H60A/R117A/R119A; “HRR→A”) point mutants substituting the conserved basic residues of the PLEKHM1 RUN domain to alanine and assessed interaction with Arl8b. Our yeast two-hybrid and dot-blot assays demonstrated that of the five conserved basic residues within the RUN domain, four (H60, R117, R119, and R123) were important for Arl8b binding (Fig. 3, a and b). As expected, the Arl8b-binding–defective mutants of PLEKHM1 had reduced overlap (∼1.5-fold decrease) with LAMP1 as compared with the WT protein (Fig. 3, c–e). We did not observe any change in the binding and colocalization of these PLEKHM1 mutants with Rab7 (Fig. 3, a, e, and g). We corroborated Arl8b binding by coimmunoprecipitation approaches as well, whereas compared with WT, no interaction of PLEKHM1 (HRR→A) was observed with Arl8b (Fig. 3 f). Importantly, PLEKHM1 (HRR→A), similar to NΔ300 and other RUN domain mutants, had an impaired ability to cluster LAMP1-positive compartments (compare the enlarged insets in Fig. 3, h and i; quantification of mean size of LAMP1-positive vesicles shown in Fig. 3 j). On the other hand, PLEKHM1 (H63A) that binds Arl8b was able to cluster LEs/lysosomes, similar to the WT (Fig. 3 j).

**Figure 4.**
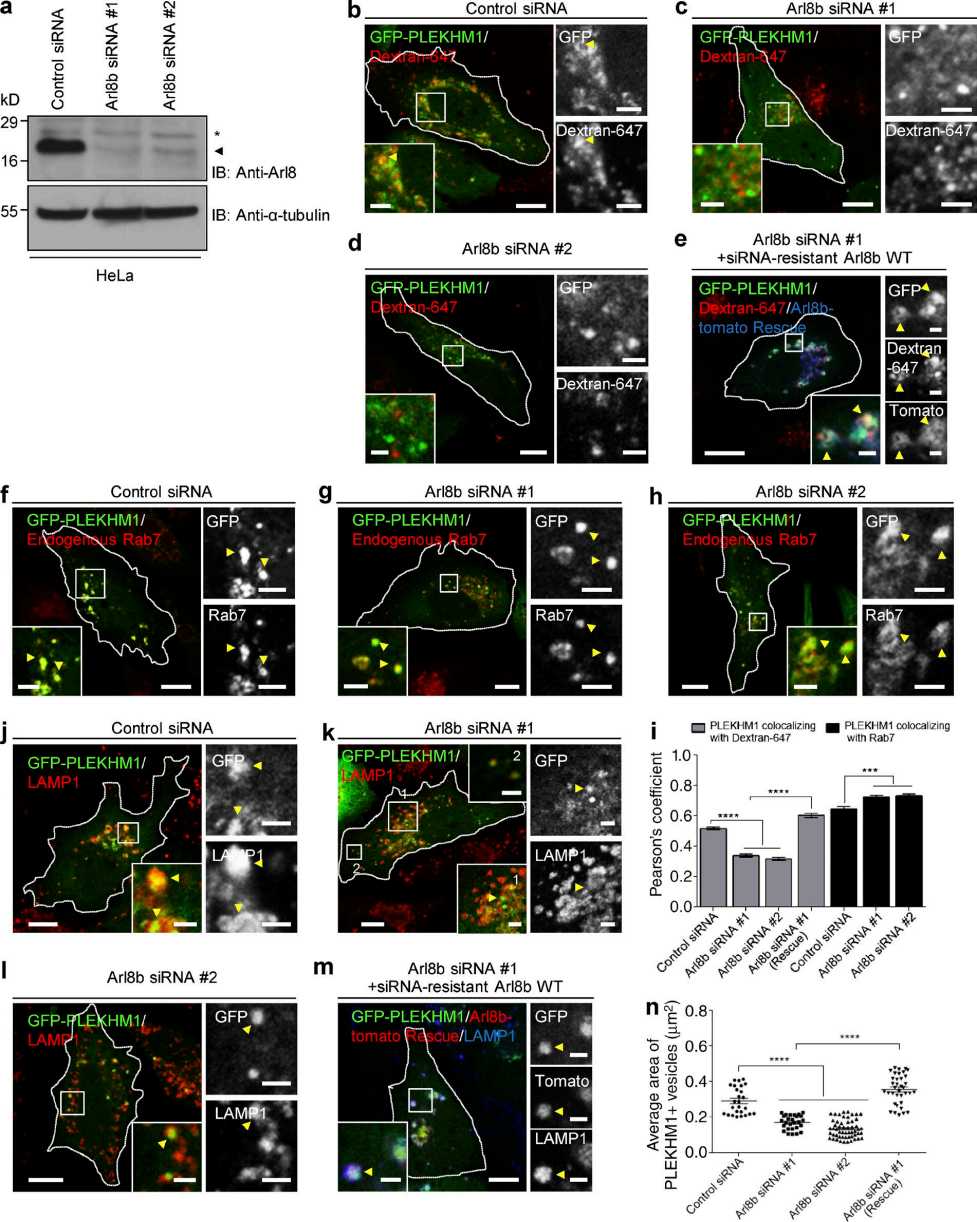
**Arl8b is required for PLEKHM1 association with lysosomes and for its ability to promote clustering of LEs and lysosomes.** (a) Control- and Arl8b-siRNA (#1 and #2)–treated HeLa cell lysates were immunoblotted (IB) with anti–Arl8 antibody for assessing the knockdown efficiency and α-tubulin as the loading control. The asterisk and arrowhead denote Arl8a and Arl8b protein bands, respectively. (b–e) Representative confocal micrographs depicting the localization of GFP-PLEKHM1 with dextran-647–loaded lysosomes in indicated siRNA treatments and Arl8b siRNA-rescued HeLa cells. Arrowheads mark colocalized pixels. (f–h) Representative confocal micrographs of HeLa cells treated with control- or Arl8b-siRNAs and transfected with GFP-PLEKHM1 followed by immunostaining for Rab7. Arrowheads mark colocalized pixels. (i) PC was calculated as a measure of colocalization of PLEKHM1 with dextran-647–loaded lysosomes or with Rab7 in control siRNA- and Arl8b siRNA-treated HeLa cells (*n* = 3; 30 cells analyzed per experiment). (j–m) Representative confocal micrographs of HeLa cells expressing GFP-PLEKHM1 and stained for LAMP1 in indicated siRNA treatments and Arl8b siRNA-rescued HeLa cells. Arrowheads mark colocalized pixels. (n) Mean size of PLEKHM1^+^ compartments in indicated siRNA treatments and Arl8b siRNA-rescued HeLa cells (*n* = 3; 10–18 cells analyzed per experiment). Data represent mean ± SEM (***, P < 0.001; ****, P < 0.0001; Student’s *t* test). Bars: (main) 10 µm; (insets) 2 µm.

Next, we depleted Arl8b from HeLa cells to directly assess its role in regulating lysosomal localization of PLEKHM1 and in PLEKHM1-mediated clustering of LEs and lysosomes. The efficiency of Arl8b depletion (using two different oligonucleotides) was >90% (Fig. 4 a). Arl8b depletion led to significantly reduced localization of PLEKHM1 to dextran-loaded lysosomes, which was rescued by siRNA-resistant Arl8b expression, suggesting that Arl8b is essential for lysosomal localization of PLEKHM1 (Fig. 4, b–e; quantification shown in Fig. 4 i). For these experiments, overnight incubation of dextran was done to ensure that it accumulates within the terminal lysosomes, which was verified by quantifying dextran colocalization with LAMP1 in control- and Arl8b-depleted cells (Fig. S2, g–i). PLEKHM1 continued to colocalize with Rab7 in Arl8b-depleted cells; rather, we observed a modest but significant increase in colocalization with Rab7 (Fig. 4, f–i). Furthermore, the mean size of PLEKHM1-positive endosomes was reduced by approximately twofold upon Arl8b depletion, which was rescued by siRNA-resistant Arl8b expression (Fig. 4, j–n). Collectively, our results suggest that Arl8b binding is required for PLEKHM1 localization to lysosomes, but not Rab7-positive LEs, and for PLEKHM1’s ability to mediate clustering of LEs and lysosomes.

### PLEKHM1 acts as a linker between the small GTPases Arl8b and Rab7

**Figure 5.**
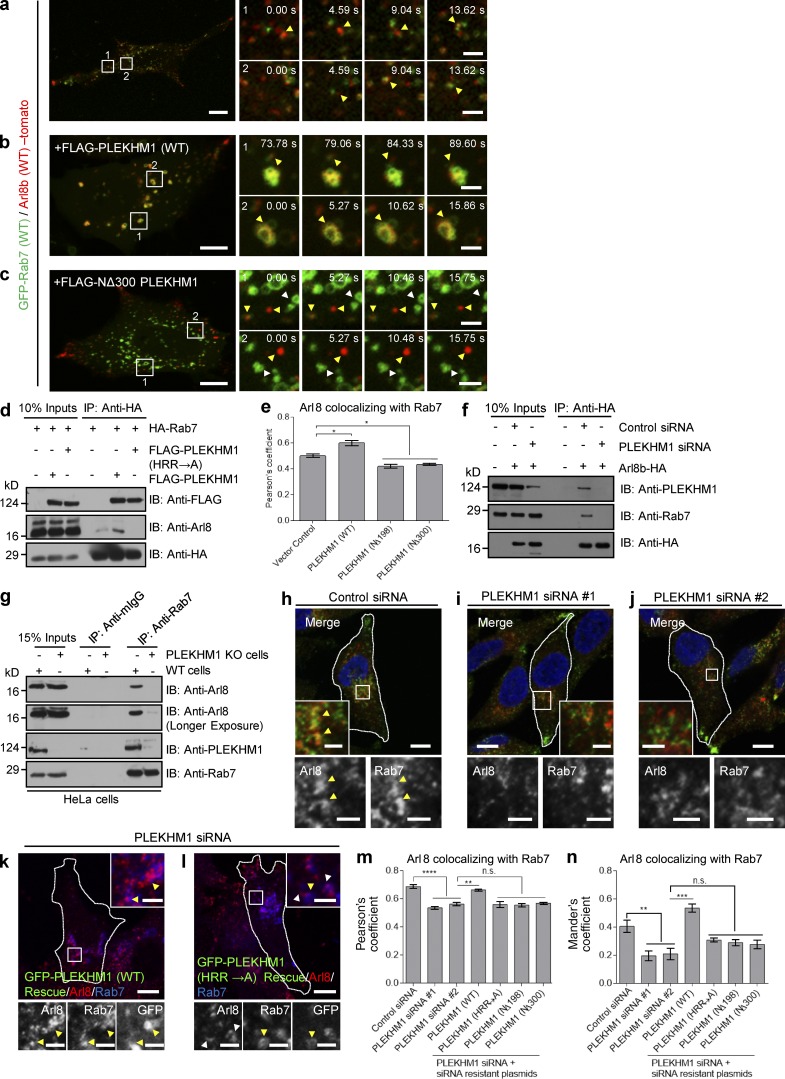
**PLEKHM1 acts as a multivalent adaptor that promotes physical interaction between Rab7 and Arl8b.** (a–c) Live-cell imaging was performed on cells expressing GFP-Rab7 and Arl8b-tomato along with either FLAG-PLEKHM1 or FLAG-NΔ300 PLEKHM1. The yellow arrowhead depicts kiss-and-run events in a and clustered enlarged endolysosomes in b, respectively. Rab7- and Arl8b-positive punctate structures that do not fuse in c are marked by white and yellow arrowheads. (d) HEK293T cell lysates expressing HA-Rab7 alone or coexpressed with either FLAG-PLEKHM1 (WT) or FLAG-PLEKHM1 (HRR→A) were immunoprecipitated (IP) with anti–HA antibody resin and immunoblotted (IB) using the indicated antibodies. (e) PC of Arl8 and Rab7 immunostained in HeLa cells transfected with indicated plasmids (*n* = 3; 30 cells analyzed per experiment). (f) Arl8b-HA was transfected in control- or PLEKHM1-siRNA–treated HEK293T cells. The lysates were immunoprecipitated with anti–HA antibody resin and immunoblotted using the indicated antibodies. (g) Lysates of WT- and PLEKHM1 KO-HeLa cells were immunoprecipitated with anti–Rab7 antibody resin and immunoblotted with the indicated antibodies. (h–j) Representative confocal images of HeLa cells treated with control siRNA or PLEKHM1 siRNAs and immunostained with anti–Arl8 and anti–Rab7 antibodies. Arrowheads mark colocalized pixels, and the nucleus was stained using DAPI. (k and l) Representative confocal micrographs of PLEKHM1 siRNA–treated HeLa cells expressing siRNA-resistant GFP-PLEKHM1 (WT) or GFP-PLEKHM1 (HRR→A) and immunostained for Arl8 and Rab7. In the insets, yellow arrowheads mark colocalized pixels. (m and n) PC and MC were calculated for Arl8 and Rab7 colocalization in indicated siRNA treatments of HeLa cells (*n* = 3; 30 cells analyzed per experiment). Data represent mean ± SEM (n.s., not significant; *, P < 0.05; **, P < 0.01; ***, P < 0.001; ****, P < 0.0001; Student’s *t* test). Bars: (main) 10 µm; (insets) 2 µm.

Our results, described thus far indicate that PLEKHM1 binds to Rab7 and Arl8b using distinct domains. To investigate whether PLEKHM1 acts as a linker between the two GTPases, we analyzed interaction of Rab7 and Arl8b in the presence and absence of PLEKHM1 using multiple approaches. In live-cell imaging experiments, we observed numerous transient kiss-and-run events between epitope-tagged Rab7 and Arl8b, which was consistent with a weak coimmunoprecipitation of Arl8b with Rab7 (and vice versa) detected in cells with physiological expression of PLEKHM1 (Fig. 5, a, d [lane 4], and f [lane 5]; and Video 1). Overexpression of WT, but not the (HRR→A) mutant of PLEKHM1, enhanced the interaction and colocalization between the two GTPases, whereas no interaction was observed upon PLEKHM1 depletion (Fig. 5, d [lanes 5 and 6], e, and f [lane 6]). This was also observed in live-cell imaging upon PLEKHM1 transfection, wherein Rab7 and Arl8b remained highly colocalized over time on the tightly clustered, less motile endolysosomes (Fig. 5 b and Video 2). Dual-color stimulated emission depletion superresolution microscopy also revealed that Arl8b and Rab7 were present on distinct regions of the same ring-shaped structures in cells with physiological expression of PLEKHM1. However, upon increased expression of PLEKHM1, Arl8b, and Rab7 showed a striking colocalized distribution on several of the enlarged and tightly clustered compartments (Fig. S2, j–m). Notably, we found a dominant-negative effect on the interaction and colocalization of Rab7 and Arl8b in cells transfected with Arl8b-binding–defective mutants of PLEKHM1 (Fig. 5, c–e; and Video 3). One possible explanation could be competition between the overexpressed mutant proteins and endogenous PLEKHM1 for binding to Rab7, thereby disrupting function of the endogenous protein.

We further tested if Rab7 and Arl8b interact at physiological expression levels. Indeed, as shown in Fig. 5 g, Arl8b was coimmunoprecipitated with Rab7, and this interaction was abrogated in a PLEKHM1-KO cell line. Notably, we also detected endogenous PLEKHM1 in complex with Rab7 and Arl8b. In accordance with our biochemical experiments, colocalization of Arl8b and Rab7 was significantly reduced upon PLEKHM1 depletion, which was rescued by siRNA-resistant PLEKHM1 (WT), but not by the Arl8b-binding–defective mutants (Fig. 5, h–n). Collectively, these results illustrate that PLEKHM1 promotes physical interaction between two key regulators of the LE/lysosome pathway, Rab7 and Arl8b.

### Arl8b is required for PLEKHM1 interaction with the multisubunit tethering factor HOPS complex

**Figure 6.**
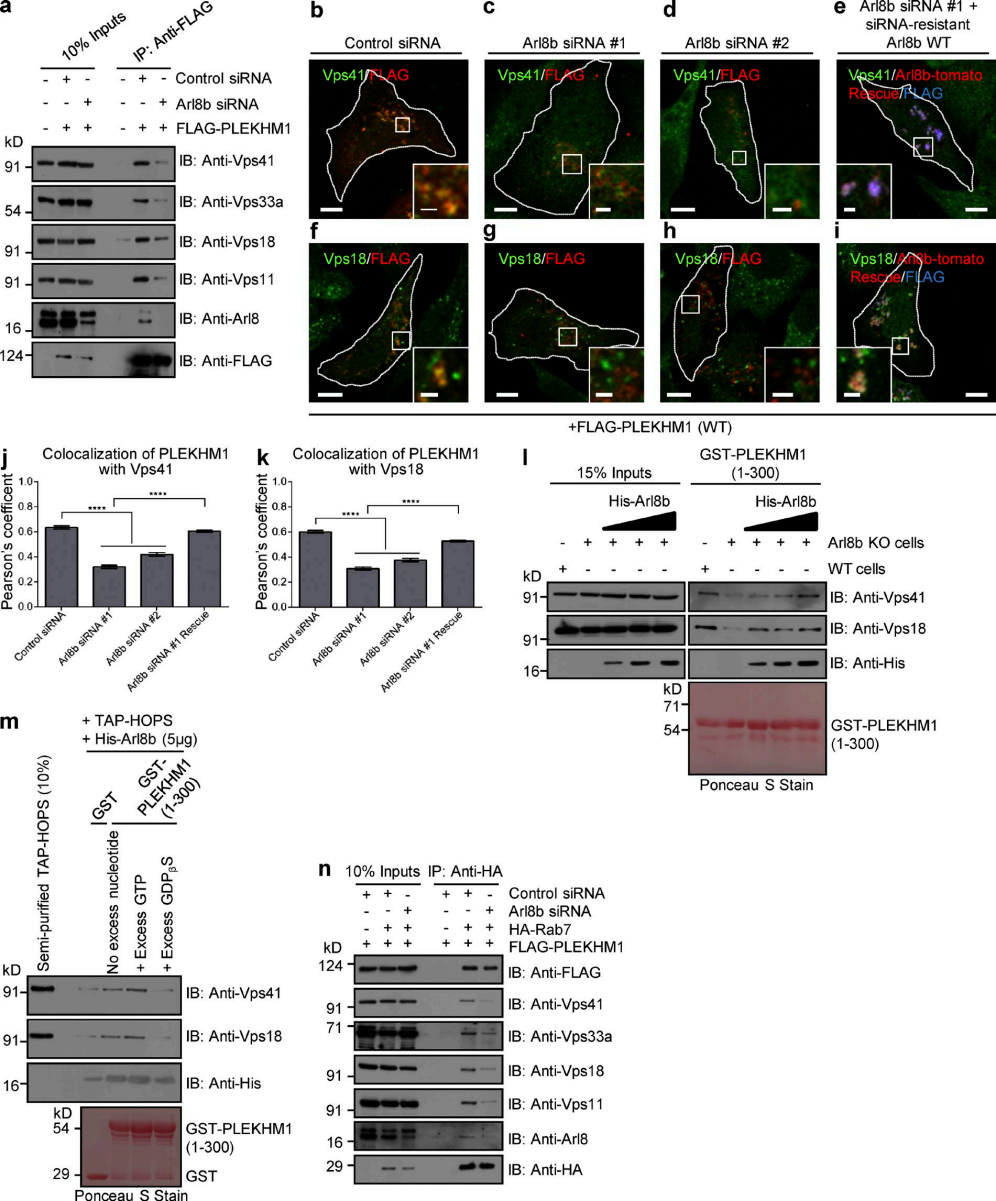
**Arl8b recruits the HOPS complex to Rab7-PLEKHM1–positive endosomes.** (a) Lysates from HEK293T cells treated with control- or Arl8b-siRNA and expressing FLAG-PLEKHM1 were IP with anti-FLAG Abs-resin and IB with indicated antibodies. (b–i) Representative confocal micrographs of HeLa cells treated with either control- or Arl8b-siRNA and expressing FLAG-PLEKHM1 (WT) alone or coexpressed with siRNA resistant Arl8b-tomato and stained for Vps41 or Vps18. (j and k) Colocalization of FLAG-PLEKHM1 with Vps41 or Vps18 was quantified by measuring PC in indicated siRNA-treated HeLa cells (*n* = 3; 30 cells analyzed per experiment). (l) Western blot of GST-pulldown assay using GST-PLEKHM1 (1–300) as bait incubated with lysates from either WT- or Arl8b KO-HeLa cells with increasing concentration of His-Arl8b protein and immunoblotted (IB) with the indicated antibodies. (m) GST-pulldown assay using semipurified TAP–HOPS complex isolated from HeLa cells incubated with either GST or GST-PLEKHM1 (1–300), His-Arl8b, and excess GTP or GDPβS. (n) Lysates of HEK293T cells treated with either control- or Arl8b-siRNA followed by cotransfection with FLAG-PLEKHM1 and HA-Rab7 were subjected to immunoprecipitation (IP) with anti–HA antibody resin and immunoblotted with the indicated antibodies. Data represent mean ± SEM (****, P < 0.0001; Student’s *t* test). Bars: (main) 10 µm; (insets) 2 µm.

PLEKHM1 has been previously shown to bind and recruit the HOPS subunits Vps41 and Vps39 to vesicle contact sites of LEs/autophagosomes and lysosomes, promoting tethering of these compartments (McEwan et al., 2015a). In yeast two-hybrid experiment, whereas we observed PLEKHM1 interaction with Vps39, no interaction was detected with Vps41. A weaker but detectable interaction was also observed with Vps18 subunit of the HOPS complex (Fig. S3 a). Evidently, PLEKHM1 (HRR→A) that was defective in LEs/lysosome clustering continued to colocalize and interact with Vps39 (Fig. S3, b and c). Surprisingly, unlike WT PLEKHM1, this mutant failed to coimmunoprecipitate HOPS complex with the exception of Vps39 (Fig. S3 d). As PLEKHM1 (HRR→A) was defective in Arl8b-binding, we hypothesized that interaction with Arl8b was required for PLEKHM1 to recruit HOPS complex. Silencing of Arl8b profoundly reduced the fraction of HOPS subunits (except Vps39) coimmunoprecipitated with PLEKHM1 (Fig. 6 a and Fig. S3 e). Consistent with this, colocalization of Vps41 and Vps18 with PLEKHM1 was reduced upon Arl8b depletion, which was rescued by siRNA-resistant Arl8b expression (Fig. 6, b–k). As expected by its direct binding, Vps39 was recruited to PLEKHM1-positive endosomes in both control and Arl8b-depleted cells (Fig. S3, f and g). RUN domain of PLEKHM1 has been previously reported to bind HOPS subunits Vps41 and Vps39 (McEwan et al., 2015a). We reasoned that direct binding of Arl8b with the RUN domain of PLEKHM1 explains these observations. Indeed, reduced binding of Vps41 and Vps18 with GST-PLEKHM1 (1–300) or (1–198) protein fragments was observed in Arl8b-KO cell lysates when compared with the WT control (Fig. 6 l and Fig. S3 h). Binding to HOPS subunits was reconstituted upon addition of increasing amounts of purified Arl8b to the cell lysates, validating that Arl8b mediates the interaction between the HOPS complex and PLEKHM1 (Figs. 6 l and S3 h). We next used a purified protein–protein interaction assay to evaluate if Arl8b directly promoted binding of HOPS subunits to the RUN domain. To this end, we isolated the HOPS complex from HeLa cell lysates using tandem affinity purification (TAP)–tagged Vps41 as bait. Mass spectrometry analysis confirmed enrichment of HOPS subunits in these eluates (Table S2). Binding of the semipurified HOPS complex was observed with GST-PLEKHM1 (1–300) or (1–198) protein fragments in the presence of GTP-bound Arl8b, but not GDP (Fig. 6 m and Fig. S3 i). These results clearly demonstrate that active Arl8b is an essential factor required for the interaction between the HOPS complex and PLEKHM1. Conversely, Arl8b’s interaction with multiple HOPS subunits was not dependent on PLEKHM1 expression (Fig. S3 j). In further support of Arl8b function in recruitment of the HOPS complex, little or no interaction of HOPS subunits was observed with Rab7 and PLEKHM1 in Arl8b-silenced cells as compared with control (Fig. 6 n).

The Rab7 effector RILP has been shown to directly bind and recruit Vps41 to Rab7/PLEKHM1-positive endosomes (van der Kant et al., 2013; Wijdeven et al., 2016). We found that whereas PLEKHM1 continued to colocalize with RILP, Vps41 recruitment to these perinuclear LEs/lysosomes was strikingly reduced in Arl8b-silenced cells (Fig. S3, k and l). These results are in agreement with our previous observations that the Rab7–RILP complex is unable to recruit Vps41 on lysosomes upon Arl8b depletion (Khatter et al., 2015a).

### Arl8b regulates PLEKHM1 function in degradation of endocytosed cargo

**Figure 7.**
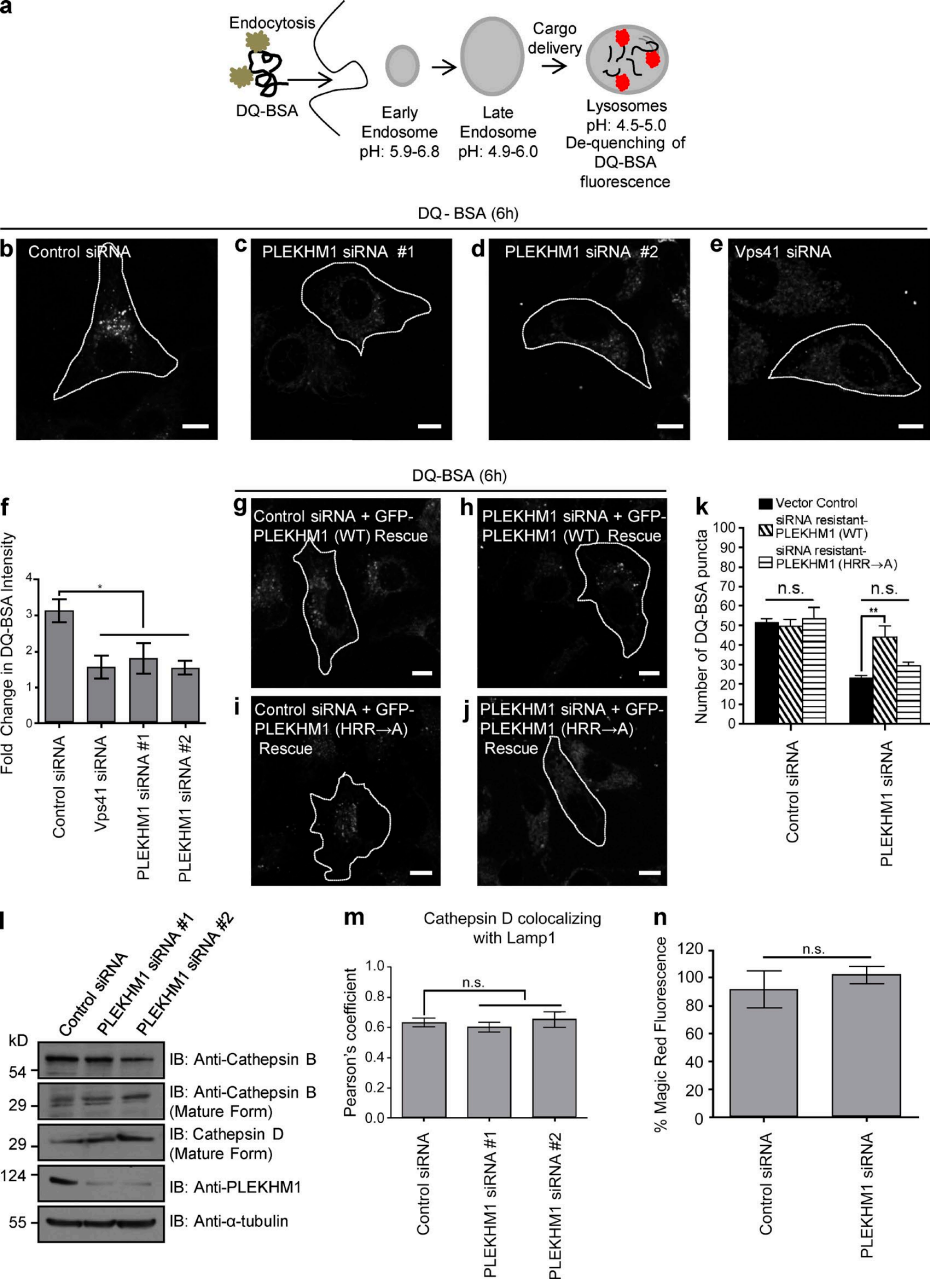
**Binding to Arl8b is necessary for PLEKHM1 function in regulating endocytic cargo trafficking to lysosomes.** (a) Schematic illustrating the uptake and further processing of DQ-BSA, an endocytic cargo in the cells. (b–e) Representative confocal images of HeLa cells treated with indicated siRNAs and subjected to DQ-BSA uptake for 6 h. The cells were then fixed and analyzed for DQ-BSA fluorescence. (f) Measurement of fold change in the fluorescence intensity of DQ-BSA from 1h to 6 h (*n* = 3; 50 cells analyzed per experiment). (g–j) Representative confocal micrographs of HeLa cells treated with the indicated siRNAs and transfected with either siRNA-resistant PLEKHM1 (WT) or siRNA-resistant PLEKHM1 (HRR→A) construct and subjected to DQ-BSA uptake for 6 h. (k) Quantification of DQ-BSA trafficking in HeLa cells treated with indicated siRNAs and transfected with either siRNA-resistant PLEKHM1 (WT) or siRNA-resistant PLEKHM1 (HRR→A) construct (*n* = 3; 50 cells analyzed per experiment). (l) Western blot of mature cathepsin B and D levels in control- or PLEKHM1-siRNA–treated HeLa cells. (m) PC was measured for cathepsin D, and LAMP1 colocalization in control- or PLEKHM1-siRNA–treated HeLa cells (*n* = 3; 30 cells per experiment). (n) HeLa cells treated with control- or PLEKHM1-siRNA were incubated for 1 h in growth medium supplemented with cathepsin L substrate, and fluorescence intensity was measured by flow cytometry (*n* = 3; 10,000 cells analyzed per experiment). Data represent mean ± SEM (n.s., not significant; *, P < 0.05; **, P < 0.01; Student’s *t* test). Bars, 10 µm.

To this point, our findings suggest that PLEKHM1 acts as a linker to promote endolysosome formation by binding to both Rab7 and Arl8b and Arl8b recruits the HOPS complex to PLEKHM1-containing endosomes. We next assessed significance of Arl8b binding in regulating PLEKHM1 function in cargo trafficking to lysosomes. To first confirm whether PLEKHM1 mediates cargo delivery to lysosomes, control- and PLEKHM1-siRNA transfected cells were incubated with DQ-BSA, an endocytic cargo that becomes fluorescent upon proteolytic cleavage in lysosomes (Fig. 7 a). The intensity of fluorescent DQ-BSA punctae in PLEKHM1-siRNA transfected cells was reduced by approximately twofold as compared with the control, suggesting that PLEKHM1 depletion impairs endolysosome fusion (Fig. 7, b–f). As a positive control for this assay, we treated cells with siRNA against Vps41, which has been previously shown to regulate endolysosome fusion (Fig. 7, e and f). Similarly, trafficking of another endocytic cargo, 3,3′-dioctadecylindocarbocyanine-low density lipoprotein (Dil-LDL), to lysosomes was impaired upon PLEKHM1 depletion (Fig. S4, a–g). To establish if Arl8b binding was required for PLEKHM1 function during endocytic cargo degradation, rescue of DQ-BSA degradation was quantified in PLEKHM1-siRNA–treated cells transfected with siRNA-resistant WT or PLEKHM1 (HRR→A) (Fig. 7, g–k). Although PLEKHM1 (WT) was able to rescue the defect in cargo degradation as indicated by an increase in DQ-BSA punctae, PLEKHM1 (HRR→A) failed to rescue this effect (Fig. 7 k), suggesting that Arl8b binding is required for PLEKHM1’s role in degradation of endocytic cargo.

To test if defective cargo degradation in PLEKHM1-depleted cells was caused by impaired lysosomal protease activity, we compared the levels of mature cathepsin B and D in control- and PLEKHM1-siRNA–treated cells. As shown in Fig. 7 l, no differences in the levels of mature cathepsin in control- and PLEKHM1-siRNA–treated cell lysates were observed. Colocalization of cathepsin D with LAMP1 was also found to be unchanged upon PLEKHM1-siRNA (Fig. 7 m). We also measured cathepsin activity by incubating control- and PLEKHM1-siRNA–treated cells with the membrane permeable probe Magic red cathepsin L substrate that emits fluorescence upon cleavage by cathepsin L. As shown in Fig. 7 n, fluorescence intensity of the cleaved cathepsin substrate was unchanged upon PLEKHM1 depletion, suggesting that PLEKHM1 regulates endocytic cargo delivery to lysosomes, but not lysosomal protease activity.

### Arl8b regulates PLEKHM1 function in autolysosome formation

**Figure 8.**
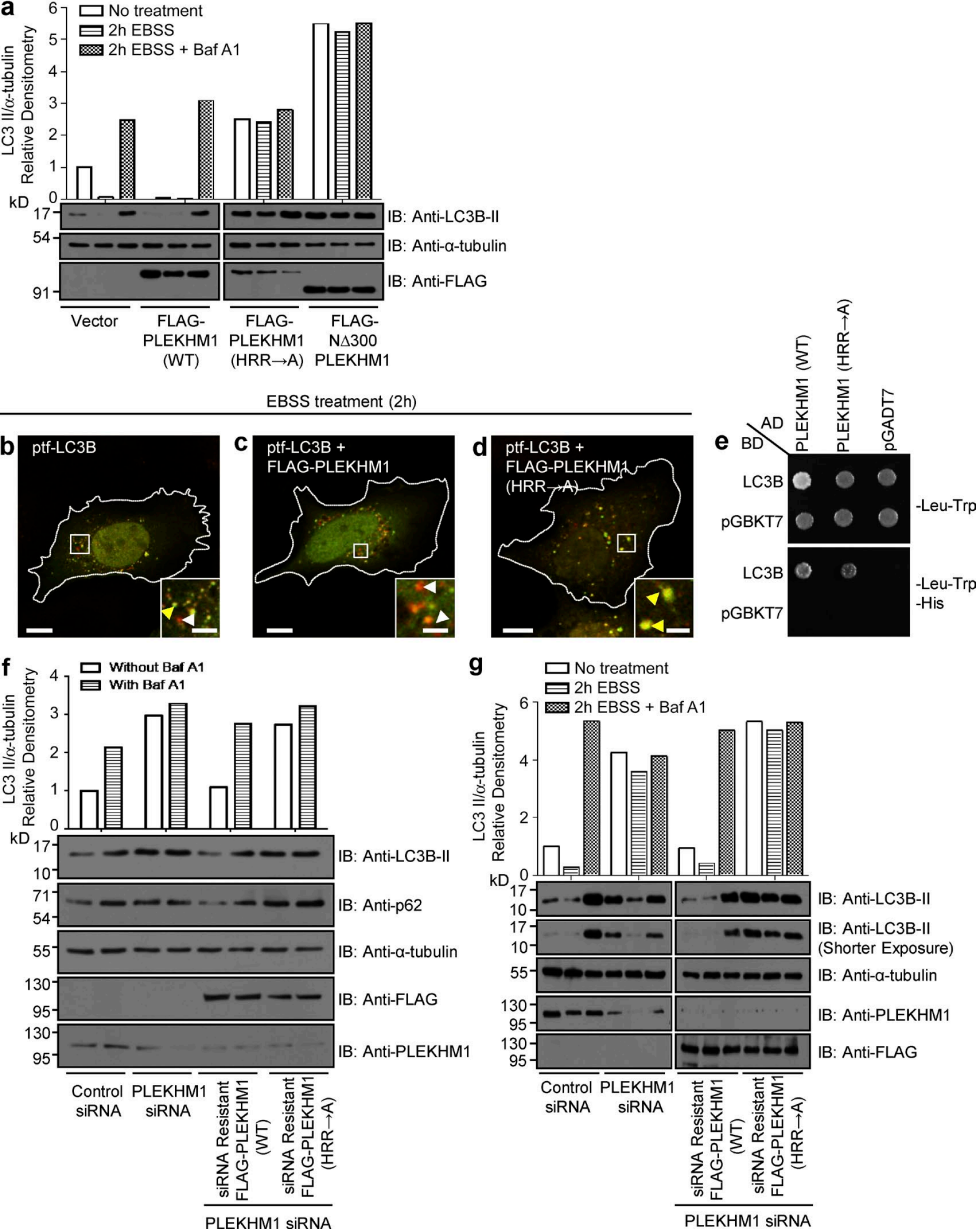
**PLEKHM1 binds Arl8b to mediate autophagosome–lysosome fusion.** (a) U2OS cells were transfected with vector alone (control), FLAG-PLEKHM1 (WT), -PLEKHM1 (HRR→A), or -NΔ300 PLEKHM1 constructs and subjected to 2 h of starvation using EBSS media in the presence or absence of Baf A1. Lysates from these cell types were immunoblotted (IB) with the indicated antibodies. (b–d) Representative confocal micrographs of HeLa cells expressing ptf-LC3B alone, cotransfected with FLAG-PLEKHM1 (WT) or FLAG-PLEKHM1 (HRR→A), and starved for 2 h in EBSS. Red-only punctate structures in magnified insets represent autolysosomes marked by white arrowheads, and yellow punctate structures represent autophagosomes marked by yellow arrowheads. (e) Yeast two-hybrid assay. Cotransformants were spotted on -Leu-Trp and -Leu-Trp-His media to confirm viability and interactions, respectively. (f) U2OS cells treated with the indicated siRNAs were given Baf A1 treatment in normal growth medium. Lysates were immunoblotted with the indicated antibodies. The levels of LC3B-II normalized to α-tubulin were quantified using densitometric analysis as shown. (g) U2OS cells treated with indicated siRNAs were further subjected to the following treatments: normal growth medium or starvation in EBSS media for 2 h with or without Baf A1. The lysates were immunoblotted for the indicated antibodies. Bars: (main) 10 µm; (insets) 2 µm.

PLEKHM1 harbors an LC3/GABARAP-interaction motif located between the two PH domains that enable it to promote clustering and fusion of LC3-positive autophagosomes with LEs/lysosomes (McEwan et al., 2015a). We hypothesized that Arl8b binding required for PLEKHM1 should be important for its function in promoting autolysosome formation. To test this, we assessed lipidated LC3 (LC3B-II) levels in nonstarved and starved U2OS cells transfected with vector alone (control), PLEKHM1 (WT), PLEKHM1 (HRR→A), and NΔ300 PLEKHM1. As depicted in Fig. 8 a, LC3B-II levels were significantly reduced upon PLEKHM1 (WT) expression compared with control (vector transfected), which was rescued upon treatment with bafilomycin A1 (Baf A1), a chemical inhibitor of autophagosome–lysosome fusion (Klionsky et al., 2016). In contrast, cells transfected with PLEKHM1 (HRR→A) and NΔ300 PLEKHM1 showed an approximately twofold and fourfold accumulation of LC3B-II levels, respectively, under both nonstarved and starved conditions with no further increase in LC3B-II levels observed upon Baf A1 treatment (Fig. 8 a). These results demonstrate the dominant-negative effect of the Arl8b-binding–defective mutants of PLEKHM1 on autolysosome formation.

We verified our observations using the tandem-fluorescence (RFP-GPF) LC3B (tfLC3B) construct, in which the acid-sensitive GFP signal is quenched at the low pH of autolysosomes but no change is observed in the acid-insensitive RFP signal (Kimura et al., 2007). Although we noted an increase in autolysosome formation in PLEKHM1 (WT) transfected cells, this effect was completely abrogated in cells expressing PLEKHM1 (HRR→A) (Fig. 8, b–d). Given that PLEKHM1 directly binds to LC3B/GABARAP proteins, we confirmed that this outcome was not caused by the lack of the PLEKHM1 (HRR→A) mutant’s ability to bind LC3B (Fig. 8 e). Finally, we rescued LC3B-II accumulation observed upon PLEKHM1 depletion with either siRNA-resistant PLEKHM1 (WT) or (HRR→A) mutant. As shown in Fig. 8 (f and g), under both nonstarved and starved conditions, there was an almost threefold increase in LC3B-II levels in PLEKHM1-siRNA transfected cells compared with the control, with no further increase observed upon treatment with Baf A1. Strikingly, unlike the siRNA-resistant PLEKHM1 (WT), the Arl8b-binding–defective mutant of PLEKHM1 was unable to rescue LC3B-II accumulation in PLEKHM1-siRNA–treated cells (Fig. 8, f and g). A similar result was obtained when we analyzed levels of the autophagy substrate p62 in these cell lysates (Fig. 8 f, second panel). Collectively, our data implicate PLEKHM1 interaction with Arl8b as a crucial factor regulating autophagosome–lysosome fusion.

### The RUN domain–containing proteins PLEKHM1 and SKIP compete for binding to Arl8b

**Figure 9.**
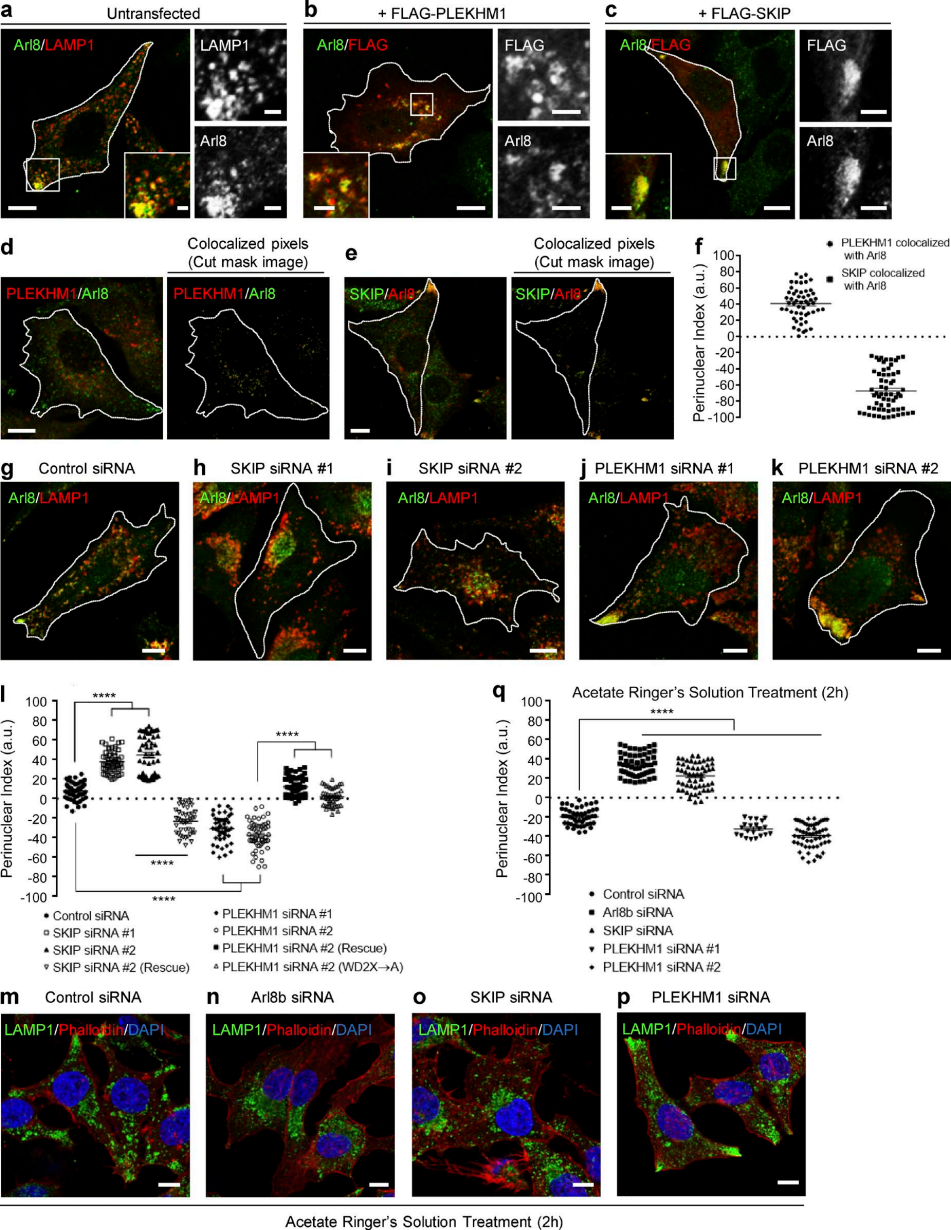
**PLEKHM1 and SKIP play opposing roles in regulating lysosome positioning.** (a–c) Representative confocal images of HeLa cells transfected with vector, FLAG-PLEKHM1, or FLAG-SKIP and immunostained for Arl8 and LAMP1. (d and e) Representative confocal images of HeLa cells immunostained for Arl8 and PLEKHM1 or SKIP. Only cut-mask image of the colocalized pixels eliminating background and individual pixels are shown on the right. (f) Perinuclear index of colocalized Arl8/PLEKHM1 or Arl8/SKIP pixels were calculated (*n* = 3; 15–20 cells analyzed per experiment). (g–k) Representative confocal images of HeLa cells treated with indicated siRNAs and stained for Arl8 and LAMP1. (l) PI of LAMP1^+^-compartments in HeLa cells transfected with indicated siRNAs and siRNA-resistant constructs (*n* = 3; 14–19 cells analyzed per experiment). (m–p) Representative confocal micrographs of HeLa cells treated with the indicated siRNAs followed by 2-h incubation in acetate Ringer’s solution, pH 6.9, and immunostained for LAMP1 to mark lysosomes. To mark the cell boundary, actin staining was performed using phalloidin and the nucleus was stained using DAPI. (q) Quantification of perinuclear index in HeLa cells treated with indicated siRNAs followed by 2-h incubation in acetate Ringer’s solution (*n* = 3; 10–18 cells analyzed per experiment). Data represent mean ± SEM (****, P < 0.0001; Student’s *t* test). Bars: (main) 10 µm; (insets) 2 µm.

Lysosomes residing in the cell periphery have attracted considerable attention for their role in various cellular processes. In accordance with its function in promoting anterograde motility of lysosomes, Arl8b is predominantly localized to the peripheral pool of lysosomes, as visualized both endogenously or when overexpressed in cells (Figs. 9 a and S5 a). Interestingly, upon transfection of PLEKHM1, Arl8b-positive lysosomes were repositioned to the perinuclear region (Fig. 9 b and Fig. 2, j and m). Although SKIP overexpression led to an opposite phenotype of Arl8b-positive lysosome accumulation at the cell periphery (Fig. 9 c). In line with these observations, we noted that under physiological conditions, PLEKHM1 and SKIP were localized to perinuclear and peripheral Arl8b-positive endosomes, respectively (Fig. 9, d–f). Next, we analyzed distribution of the Arl8b/LAMP1-positive endosomes in PLEKHM1- and SKIP-depleted cells. As previously reported (Rosa-Ferreira and Munro, 2011), siRNA-mediated knockdown of SKIP resulted in the clustering of Arl8b/LAMP1-positive compartment in the perinuclear region (Fig. 9, h and i; quantification shown in Fig. 9 l). PLEKHM1 depletion, on the other hand, led to a striking accumulation of Arl8b/LAMP1-positive endosomes at the cell periphery (Fig. 9, j–l). Lysosomal distribution was restored by transfection of the siRNA-resistant constructs (SKIP and PLEKHM1) in respective siRNA-treated cells (Fig. 9 l).

It has been previously shown that a reduction in cytoplasmic pH drives anterograde motility of lysosomes in Arl8b- and SKIP-dependent manner (Rosa-Ferreira and Munro, 2011). We found a similar dramatic decrease in the acid-induced peripheral pool of lysosomes upon depletion of either SKIP or Arl8b (Fig. 9, m–o; quantification shown in Fig. 9 q). In contrast, a significant increase in the acid-induced peripheral pool of lysosomes was observed in PLEKHM1-depleted cells (Fig. 9, p and q). These results clearly demonstrate that PLEKHM1 and SKIP have opposing effects on lysosomal distribution. Although we cannot rule out that the lysosomal levels of the retrograde motor protein dynein are reduced upon PLEKHM1 depletion, we found that overexpression of RILP, an adaptor that recruits the dynein–dynactin complex to lysosomes, completely reversed the peripheral lysosomal accumulation observed in PLEKHM1-siRNA–treated cells (Fig. S5, b and c). These data indicate that the dynein–dynactin complex is most likely recruited to lysosomes upon PLEKHM1 depletion.

**Figure 10.**
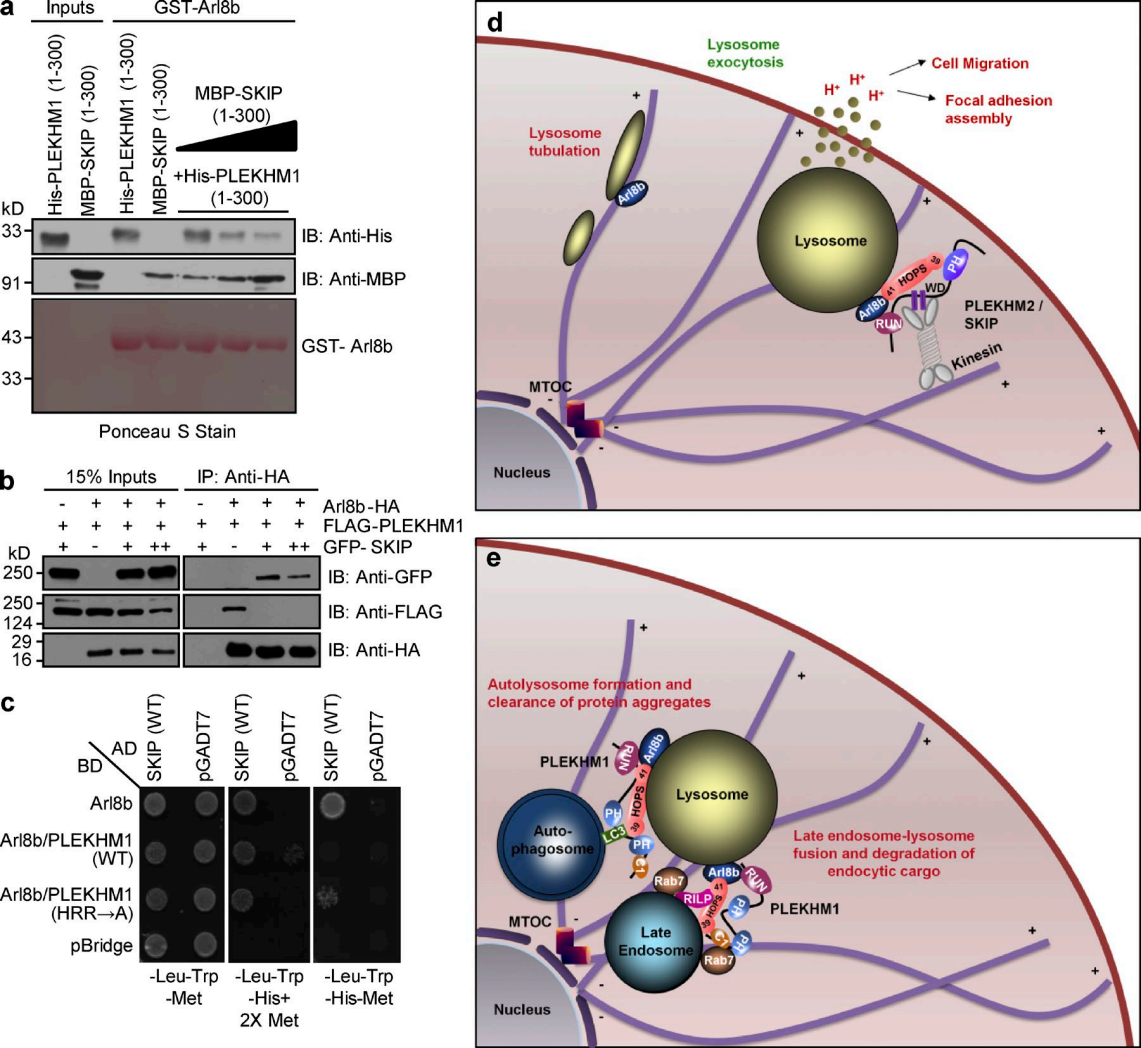
**PLEKHM1 and SKIP compete for binding to Arl8b via their respective RUN domains.** (a) Immunoblot (IB) of competition assay done using GST-Arl8b as bait and incubated with His-PLEKHM1 (1–300) and increasing concentrations of MBP-SKIP (1–300). (b) Immunoblot of an immunoprecipitation (IP) assay using HEK293T cells lysates coexpressing Arl8b-HA and FLAG-PLEKHM1 with increasing amounts of GFP-SKIP. (c) Yeast three-hybrid assay. Cotransformants were spotted on -Leu-Trp-Met medium to check for viability and on -Leu-Trp-His+2X Met and -Leu-Trp-His-Met media to test the interaction and competition, respectively. (d and e) Proposed model of lysosome distribution and function regulation by small GTPase Arl8b and its effectors, PLEKHM1 and SKIP. SKIP interacts with Arl8b via its RUN domain, further recruiting kinesin motor that drives anterograde lysosome motility, which is implicated in regulating cellular processes like cell migration/invasion and focal adhesion assembly. Here, we report PLEKHM1 as a dual effector of Rab7 and Arl8b that simultaneously binds these GTPases, bringing about clustering and fusion of LEs and lysosomes. PLEKHM1 also binds to LC3 and promotes autolysosome formation.

Our results led us to hypothesize that the RUN domain–containing proteins SKIP and PLEKHM1 compete for binding to Arl8b, which in turn regulates lysosome positioning. In support of this hypothesis, we found that arginine residues within the block D of the conserved core of the SKIP RUN domain (R92 and R94; residues shown in Fig. S2 f) were required for binding to Arl8b (Fig. S5, d–f). To test whether SKIP competes with PLEKHM1 for binding to Arl8b, we performed a purified protein–protein interaction assay where increasing amounts of MBP-tagged SKIP (1–300) were able to outcompete His-tagged PLEKHM1 (1–300) for binding to GST-Arl8b (Fig. 10 a). Additionally, as shown in Fig. S5 g and Fig. 10 b, transfection with SKIP (1–300) or full-length construct resulted in loss of PLEKHM1 binding to Arl8b. We also used a yeast three-hybrid assay to test the interaction of Arl8b and SKIP in the presence of either PLEKHM1 (WT) or PLEKHM1 (HRR→A) mutant. In this assay PLEKHM1 (WT) and PLEKHM1 (HRR→A) expression was under the control of the Met25 promoter, which is repressed in the presence of methionine (Met) in the growth media. As depicted in Fig. 10 c, under Met-deficient conditions, Arl8b’s interaction with SKIP was abrogated in the presence of PLEKHM1 (WT), but not upon expression of the PLEKHM1 (HRR→A) mutant, suggesting that SKIP and PLEKHM1 compete with each other for Arl8b binding. To test if altered lysosomal distribution in PLEKHM1-siRNA–treated cells results from an increased binding of Arl8b to SKIP, we created a dominant-negative mutant of SKIP (WD2X→A) that has been previously shown to bind Arl8b, but not kinesin-1 (Rosa-Ferreira and Munro, 2011). We confirmed these findings by visualizing recruitment of kinesin light chain (KLC2) in cells expressing WT or (WD2X→A) SKIP mutant along with Arl8b (Fig. S5, h and i). Notably, (WD2X→A) SKIP mutant partially reversed the peripheral lysosomal distribution observed in PLEKHM1-siRNA–treated cells, suggesting that increased association of SKIP with Arl8b drives lysosomes to the cell periphery upon PLEKHM1 depletion (Fig. 9 l).

## Discussion

Rab7 and Arl8b are central players that orchestrate microtubule-dependent transport of LEs/lysosomes and their fusion with endosomes, autophagosomes, and phagosomes. Despite an overlapping subcellular localization, and similar roles in membrane trafficking, it was not known whether there is cross talk between Rab7 and Arl8b to coordinate their functions. Here, we have identified a role for the Rab7 effector PLEKHM1 as a dual effector of Arl8b to promote cargo delivery to lysosomes. Whereas Rab7 is required for membrane recruitment of PLEKHM1, our study shows that Arl8b regulates PLEKHM1 lysosomal localization and its ability to promote clustering of the late endosomal/lysosomal compartments. Our findings suggest that PLEKHM1 requires coordinated activation of both Rab7 and Arl8b to function as an adaptor in the endolysosomal pathway.

PLEKHM1 has been previously reported to recruit subunits of the HOPS complex (Vps39 and Vps41) and the associated SNARE protein (syntaxin 17) to the vesicle–lysosome contact sites (McEwan et al., 2015a). Intriguingly, we found that Vps39 binding was not sufficient for PLEKHM1 to recruit other subunits of the HOPS complex and to promote tethering of the endolysosomal compartment. These observations are in agreement with a recent study where PLEKHM1 was found to be sufficient for Vps39, but not Vps41, recruitment to LEs/lysosomes (Wijdeven et al., 2016). Our results suggest that GTP-bound Arl8b is required for pulldown of the HOPS complex with PLEKHM1 and recruitment to PLEKHM1-positive vesicle contact sites. Accordingly, a PLEKHM1 mutant that did not interact with Arl8b but continued to interact with Rab7 and Vps39 failed to rescue endocytic cargo degradation and autolysosome formation upon PLEKHM1 depletion. Based on the previous studies and our current findings, we propose a model of sequential assembly of the vesicle fusion machinery at LE/lysosome contact sites wherein the Rab7–RILP complex binds to and recruits PLEKHM1 from cytosol to perinuclear LEs. PLEKHM1, via its RUN domain, interacts with Arl8b present on lysosomes, acting as a linker between the two GTPases. In this context, it is interesting to note that PLEKHM1 repositions Arl8b-positive endosomes to the perinuclear region, which could increase their accessibility to the material from the biosynthetic pathway (Johnson et al., 2016). Arl8b then recruits Vps41 and other subunits (except Vps39) of the HOPS complex to the vesicle–lysosome contact sites, whereas Vps39 is recruited by its direct binding to PLEKHM1. This in turn promotes tethering and SNARE-mediated fusion of cargo vesicles with lysosomes (Fig. 10 e). At present, there are several important questions that remain to be answered. For instance, does Arl8b-mediated HOPS assembly on lysosomes facilitate a structural change that is required for binding to PLEKHM1, or does Arl8b act as a physical linker to mediate Vps41 binding to PLEKHM1? Although we did not observe competition between PLEKHM1 and Vps41 for binding to Arl8b, it is unlikely that these two effectors bind to a single molecule of Arl8b. Because PLEKHM1 can also potentially bridge LE/lysosome compartments by its direct affinity for Rab7 and Arl8b, could it act as a tether to promote vesicle docking with lysosomes? Taking the yeast vacuole fusion pathway as a paradigm, in vitro tethering and fusion assays will be required to determine the precise hierarchy of these interactions and comprehensively decipher the role of these two GTPases and their multitude of effectors in the heterotypic fusion of lysosomes with other compartments.

Arl8b-mediated lysosome positioning at the cell periphery regulates diverse cellular processes, including amino acid sensing, antigen presentation, cell migration, and cancer metastasis (Garg et al., 2011; Korolchuk et al., 2011; Schiefermeier et al., 2014; Dykes et al., 2016). Our study indicates that Arl8b effectors- SKIP and PLEKHM1 play opposing roles in regulating lysosome distribution. Indeed, several lines of evidences suggest that the two RUN domain-containing proteins compete for binding to Arl8b, explaining their antagonistic effect on lysosome distribution. We speculate that although Arl8b–PLEKHM1 interaction is required for cargo delivery to lysosomes, interaction with SKIP might regulate ascribed roles of lysosomes at the cell periphery, including exocytosis, cell migration, and plasma membrane repair (Fig. 10, d and e). Here, it is interesting to note that besides Arl8b, the HOPS subunit Vps39 also interacts with both SKIP and PLEKHM1, where binding to SKIP promotes Vps39 recruitment to peripheral lysosomes (Fig. 10 d; Khatter et al., 2015a). Whether SKIP and PLEKHM1 regulate positioning of the HOPS complex to peripheral or perinuclear LEs/lysosomes and the role of the HOPS complex on peripheral lysosomes needs to be investigated. In line with this, a recent study has shown that Vps39, along with Rab2a, controls exocytosis of late endosomal MT1-MMP, an essential metalloprotease required for extracellular matrix remodeling and tumor invasion (Kajiho et al., 2016).

Arl8b and PLEKHM1 interaction might have implications in the human disease osteopetrosis, a genetic disorder caused by loss-of-function mutations in *PLEKHM1* that disrupt osteoclast function in bone resorption, resulting in disorganized bone structure (Van Wesenbeeck et al., 2007). An important question therefore will be to elucidate whether Arl8b–PLEKHM1 interaction is required for bone remodeling function of osteoclasts and whether Arl8b regulates lysosome secretion in osteoclasts.

In summary, PLEKHM1 is a dual Rab/Arl effector that binds to Rab7 and Arl8b and orchestrates assembly of the vesicle fusion machinery, leading to lysosomal degradation of cargo internalized via endocytic and autophagic pathways.

## Materials and methods

### Cell culture and RNAi

HeLa, HEK293T, and U2OS (from ATCC) were cultured in DMEM (Lonza) supplemented with 10% FBS (Gibco) at 37°C with 5% CO_2_ in a humidified cell culture chamber. Each cell line was regularly screened for absence of mycoplasma contamination by using MycoAlert Mycoplasma Detection kit (Lonza) and was passaged for no more than 15 passages. For gene silencing, siRNA oligos were purchased from GE Healthcare and prepared according to the manufacturer’s instructions. Sequences of siRNA oligos used in this study were as follows: control, 5′-TGGTTTACATGTCGACTAA-3′; Arl8b, 5′-AGGTAACGTCACAATAAAGAT-3′ (siRNA #1) and 5′-GCTGAAGATGAATATCCCTAA-3′ (siRNA #2); PLEKHM1, 5′-CCGGTCTCTGCAAGAGGTATTGT-3′ (siRNA #1), 5′-GGTCTGAAGCTGGTAGTTT-3′ (siRNA #2), and 5′-GCAAAGTCCTGGCATCCTA-3′ (siRNA #3); Vps41, 5′-TGACATAGCAGCACGCAAA-3′; and SKIP, 5′-CTTCTGAACTGGACCGATT-3′.

### Generation of Arl8b and PLEKHM1 KO cells by CRISPR/Cas9

Arl8b and PLEKHM1 KO HeLa cells were generated using the Arl8b sg/RNA (5′-target sequence: GATGGAGCTGACGCTCG-3′) and PLEKHM1 sg/RNA (target sequence: 5′-GAAGCTGGTGGGATCCGTGA-3′) CRISPR/Cas9 All-in-One Lentivector Set, respectively (human; Applied Biological Materials). In brief, All-in-One plasmid was transfected into HEK293T cells together with lentiviral packaging plasmids for producing viral particles using X-tremeGENE HP DNA Transfection Reagent (Roche). Culture supernatants were harvested 48 h posttransfection, centrifuged, and concentrated using Lenti-X concentrator (Takara Bio Inc.). HeLa cells were infected with supernatants containing lentiviral particles in the presence of 8 µg/ml polybrene (Sigma-Aldrich). Lentiviral-infected cells were selected by 3 µg/ml puromycin (Sigma-Aldrich) for 72 h and then reseeded in 96-well plates to allow single-colony formation. The identification of the KO cell clones was confirmed by immunoblot analysis.

### Mammalian expression constructs

All the expression plasmids used in this study are listed in Table S1.

### Antibodies and chemicals

The following antibodies were used in this study: mouse anti-FLAG M2 clone (F1804; Sigma-Aldrich), rat anti-HA clone 3F10 (11867423001; Roche), rabbit anti-HA (sc-805; Santa Cruz Biotechnology, Inc.), mouse anti-His (SAB1305538; Sigma-Aldrich), mouse anti-MBP (E8038S; New England Biolabs, Inc.), mouse anti–α-tubulin (T9026; Sigma-Aldrich), mouse anti-HA (MMS-101P; Covance), rabbit anti-GFP (ab6556; Abcam), mouse anti-GFP (sc-9996; Santa Cruz Biotechnology, Inc.), rabbit anti-rat (ab6703; Abcam), mouse anti-Arl8 clone H-8 (sc-398635; Santa Cruz Biotechnology, Inc.), mouse anti-LAMP1 (555798; BD), mouse anti-EEA1 (610457; BD), rabbit anti-LAMP1 (ab24170; Abcam); rabbit anti-Giantin (ab80864; Abcam), rabbit anti-Cathepsin D (K50161R; Meridian Life Sciences), mouse anti-Cathepsin B clone 4B11 (414800; Thermo Fisher Scientific), rabbit anti-PLEKHM1 (ab171383; Abcam), rabbit anti-SKIP/PLEKHM2 (HPA032304; Sigma-Aldrich), mouse anti-Rab7 clone B-3 (sc-376362; Santa Cruz Biotechnology, Inc.), and rabbit anti-Rab7 (9367; Cell Signaling Technology). For detection of HOPS subunit, the following antibodies were used: rabbit anti-Vps11 (ab125083; Abcam), rabbit anti-Vps18 (ab178416; Abcam), rabbit anti-Vps33a (16896–1-AP; ProteinTech), rabbit anti-Vps41 (ab181078; Abcam), and mouse anti-Vps41 (sc-377271; Santa Cruz Biotechnology, Inc.). For autophagy-related experiments, rabbit anti–LC3B-II (3868) and rabbit anti–p62 (8025) antibodies were purchased from Cell Signaling Technology. Rabbit anti–PLEKHM1 antibody generated against the N-terminal 497 aa of human PLEKHM1 protein was a gift from P. Odgren (University of Massachusetts Medical School, Worcester, MA) and has been previously used to detect PLEKHM1 by immunofluorescence and Western blotting (Witwicka et al., 2015). Rabbit anti–Arl8 antibody used in this study has been described previously (Garg et al., 2011; Khatter et al., 2015a). All the Alexa fluorophore–conjugated secondary antibodies were purchased from Thermo Fisher Scientific. HRP-conjugated goat anti-mouse and goat anti-rabbit were purchased from Jackson ImmunoResearch Laboratories, Inc. Protein A gold for immunolabeling was purchased from University Medical Center (Utrecht, Netherlands). Phalloidin, Alexa Fluor 647–conjuated Dextran, DQ-BSA, and DAPI were purchased from Invitrogen. Earle’s Balanced Salt Solution (EBSS) and Baf A1 were purchased from Sigma-Aldrich. The Magic Red Cathepsin L Assay kit to monitor activity of the pH-sensitive protease cathepsin L was purchased from ImmunoChemistry Technologies.

### Transfections, immunofluorescence, and live-cell imaging

Cells grown on glass coverslips were transfected with desired constructs using X-tremeGENE-HP DNA transfection reagent (Roche) for 16–18 h. Cells were fixed in 4% PFA in PHEM buffer (60 mM Pipes, 10 mM EGTA, 25 mM Hepes, and 2 mM MgCl_2_, final pH 6.8) for 10 min at room temperature. Postfixation, cells were incubated with blocking solution (0.2% saponin + 5% FBS in PHEM buffer) at room temperature for 30 min, followed by three washes with 1X PBS. After this blocking step, cells were incubated with primary antibodies in staining solution (PHEM buffer + 0.2% saponin) for 45 min to 1 h at room temperature, washed thrice with 1X PBS, and further incubated for 30 min with Alexa fluorophore–conjugated secondary antibodies made in staining solution. Cells were washed thrice with 1X PBS and mounted in Fluoromount G (SouthernBiotech). Single-plane confocal images were acquired using a 710 Confocal Laser Scanning Microscope (ZEISS) equipped with a Plan Apochromat 63×/1.4 NA oil immersion objective and high-resolution microscopy monochrome cooled camera AxioCam MRm Rev. 3 FireWire (D) (1.4 megapixels, pixel size 6.45 µm × 6.45 µm). For image acquisition, ZEN Pro 2011 (ZEISS) software was used. All images of control and gene-specific siRNA or comparison of PLEKHM1 with different markers were captured at same laser gain and intensity values. All images were captured to ensure that little or no pixel saturation is observed. For quantification, images were imported into ImageJ software. The representative confocal images presented in figures were imported into Adobe Photoshop CS and formatted to 300 dpi resolution. The whole image adjustment of brightness was done using curves function. The representative images of different treatments (control versus gene-specific siRNA) were subjected to same brightness adjustments.

For detecting endogenous staining of PLEKHM1, PLEKHM2/SKIP, Arl8, and Rab7, the primary and secondary antibodies were made in PBS, pH 7.4, containing 0.05% Tween-20 + 0.5% BSA. For acetate Ringer’s solution treatment, cells were incubated with acetate Ringer’s solution (80 mM NaCl, 70 mM sodium acetate, 5 mM KCl, 2 mM CaCl_2_, 1 mM MgCl_2_, 2 mM NaH_2_PO_4_, 10 mM Hepes, and 10 mM glucose, final pH 6.9) for 2 h. Post-incubation cells were fixed, stained, and processed for confocal imaging as described in the previous paragraph.

For live-cell imaging, cells were seeded on glass-bottom tissue culture treated cell imaging dish (Eppendorf) and transfected with the indicated plasmids. Posttransfection (16–18 h), imaging dish was loaded into a sealed live-cell imaging chamber (37°C and 5% CO_2_) for imaging in DMEM. Time-lapse confocal images were acquired every 2 s using an LSM 710 confocal microscope with a LCI Plan Neofluar objective 63×/1.3 multi-immersion correction and equipped with a high-resolution microscopy monochrome cooled camera AxioCam MRm Rev. 3 FireWire (D). Image acquisition was controlled by ZEN Pro 2011 software, and adjustments to brightness and contrast were performed with ImageJ software.

### Structured illumination microscopy (SIM) and stimulated emission depletion (STED) microscopy

SIM imaging was performed at the Advanced Microscopy Core Facility at the University of Nebraska Medical Center, and the samples were processed as previously described (Reinecke et al., 2015). In brief, cells were fixed and immunostained with appropriate antibodies as described for confocal microscopy. SIM images were collected with a ZEISS ELYRA PS.1 illumination system using a 63× oil objective lens with a numerical aperture of 1.4 at room temperature. Three orientation angles of the excitation grid were acquired for each Z plane, with Z spacing of 110 nm between planes. SIM processing was performed with the SIM module of the Zen black software (ZEISS).

STED microscopy was performed at the Indian Institute of Science Education and Research Pune Leica Micro Imaging Center. Images were acquired on a Leica Biosystems TCS STED 3× microscope with a 100× 1.4 NA oil STED white objective. Alexa 594 and Alexa 647 fluorophores were excited with white light laser 561-nm and white light laser 647-nm lasers, respectively, and a 775-nm pulsed laser was used for depletion. Spectral hybrid detectors with 45% quantum efficiency were used for image acquisition in sequential mode. Images were deconvolved using the Huygens (SVI) deconvolution algorithm of the Leica Biosystems LASX software. The size of pixels used for imaging was 24 nm with an image format of 1,200 × 1,200 pixels and 4× optical zoom.

### Colocalization analysis

For all the colocalization analysis, 25–30 cells per experiment for each treatment were used for three independent experiments. Pearson’s correlation coefficient (PC; for Fig. 2 h, see below) and Mander’s coefficient (MC; Fig. S1 n) were determined using the JACoP plugin of ImageJ. PC was calculated on the original image where no threshold settings (manual or automatic) were applied. In Fig. 2 h, another method (Costes’ approach) to calculate PC was used, as the classical PC is highly sensitive to the intensity values of the two channels and to the background noise parameters that will be different when comparing colocalization of single protein with multiple markers (Costes et al., 2004). In the Costes’ method, automated and unbiased threshold is calculated by determining PC at different intensities. The final threshold is set to values that minimize the contribution of noise (i.e., PC under the threshold being negative). Further image randomization (200 times) is done, and the PC is calculated each time between the random image of one channel and the original of the other. Comparison of PCs from nonrandomized and randomized images gives the significance (p-value) of colocalization. The p-value in Fig. 2 h was 100%, suggesting that the colocalization was highly probable. To calculate the MC of endogenous proteins PLEKHM1, Rab7, and Arl8b, threshold values were set by first determining where the estimated background signal is negligible or zero. This was determined by quantification of images from control and gene-specific siRNA-treated cells. At the threshold value, negligible or no punctae in the siRNA-treated cells were highlighted. The same threshold settings were uniformly applied to all images within each experiment. Intensity threshold of 45–55 (value range is from 0 to 255) was selected for endogenous PLEKHM1, which highlighted punctate structures in WT or control siRNA-treated cells. At this threshold value, no punctate signal was highlighted in PLEKHM1-depleted cells. Similarly, a threshold of 35–40 was set for endogenous Rab7 and a threshold of 30 was defined for endogenous Arl8. As little or no background was observed upon immunostaining of EEA1/LAMP1, the threshold settings for these markers was determined where all endosomal punctae were highlighted. The same threshold settings were uniformly applied to all images within each experiment.

### Quantification of particle size and perinuclear index

For measuring particle size of LAMP1- or PLEKHM1-positive compartments, the Analyze Particles function of ImageJ was applied, where “MaxEntropy” threshold was used. To measure the particle size of DQ-BSA punctae, the Analyze Particles function of ImageJ software was applied, where “Default” threshold was used. Lysosome distribution was assessed as a measure of perinuclear index as previously described (Li et al., 2016). In brief, the fluorescence intensity of LAMP1 staining was measured in the whole cell (I_total_), the nuclear region (i.e., the area within 5 µM of nucleus; I_perinuclear_), and an area >10 µm from the nucleus (I_peripheral_). The peripheral and perinuclear intensities were calculated and normalized as I_>10_ = I_peripheral_/I_total_ − 100 and I_<5_ = I_perinuclear_/I_total_ − 100. The perinuclear index was calculated as I_<5_ − I_>10_ × 100.

### Immunogold EM

Sample processing and immunogold labeling was performed at the Harvard Medical School EM Facility. For preparation of cryosections, HeLa cells cotransfected with Arl8b-HA and GFP-PLEKHM1 were fixed with 4% PFA + 0.1% glutaraldehyde prepared in 0.1 M sodium phosphate buffer, pH 7.4. After 2-h fixation at room temperature, the cell pellet was washed once with PBS and then placed in PBS containing 0.2 M glycine for 15 min to quench free aldehyde groups. Before freezing in liquid nitrogen, the cell pellets were cryoprotected by incubating in three drops of 2.3 M sucrose in PBS for 15 min. Frozen samples were sectioned at −120°C, and the sections were transferred to formvar/carbon-coated copper grids. Grids were floated on PBS until the immunogold labeling was performed.

The double immunogold labeling was performed at room temperature on a piece of parafilm. All the primary antibodies and Protein A immunogold were diluted in 1% BSA in PBS. In brief, grids were floated on drops of 1% BSA for 10 min to block for unspecific labeling, transferred to 5-µl drops of rat anti-HA, and incubated for 30 min. The grids were then washed in four drops of PBS for a total of 15 min, transferred to 5-µl drops of rabbit anti-rat for 30 min, and washed again in four drops of PBS for 15 min, followed by 15 nm Protein A immunogold for 20 min (5-μl drops). After the 15-nm Protein A immunogold incubation, grids were washed in four drops of PBS, fixed for 2 min with 0.5% Glu followed by four drops of PBS containing 0.2 M glycine for 15 min to quench free aldehyde groups. The labeling process was repeated with rabbit anti-GFP followed by 10 nm Protein A immunogold for 20 min in 5-μl drops. Finally, the grids were washed in four drops of PBS and six drops of double-distilled water. Contrasting/embedding of the labeled grids was performed on ice in 0.3% uranyl acetate in 2% methyl cellulose for 10 min. Grids were picked up with metal loops, and the excess liquid was removed by blotting with a filter paper and were examined in an electron microscope (1200EX; JEOL). Images were recorded with an AMT 2k CCD camera.

### Coimmunoprecipitation and immunoblotting

HEK293T cells transfected with indicated plasmids were lysed in TAP lysis buffer (20 mM Tris, pH 8.0, 150 mM NaCl, 0.5% NP-40, 1 mM MgCl_2_, 1 mM Na_3_VO_4_, 1 mM NaF, 1 mM PMSF, and protease inhibitor cocktail; Sigma-Aldrich). The lysates were incubated with indicated antibody conjugated-agarose beads at 4°C rotation for 3 h, followed by four washes in TAP wash buffer (20 mM Tris, pH 8.0, 150 mM NaCl, 0.1% NP-40, 1 mM MgCl_2_, 1 mM Na_3_VO_4_, 1 mM NaF, and 1 mM PMSF). The samples were then loaded on SDS-PAGE for further analysis. Protein samples separated on SDS-PAGE were transferred onto polyvinylidene fluoride membranes (Bio-Rad Laboratories). Membranes were blocked overnight at 4°C in blocking solution (10% skim milk in 0.05% PBS-Tween 20). Indicated primary and secondary antibodies were prepared in 0.05% PBS-Tween 20. The membranes were washed for 10 min thrice with 0.05% PBS-Tween 20 or 0.3% PBS-Tween 20 after 2-h incubation with primary antibody and 1-h incubation with secondary antibody, respectively. The blots were developed using a chemiluminescence-based method.

### TAP and mass spectrometry

For semipurification of the HOPS complex from HeLa cells, TAP was performed using an InterPlay Mammalian TAP system (Stratagene). In brief, 25 million HeLa cells stably expressing N-terminal TAP-tagged Vps41 were lysed following the manufacturer’s protocol. TAP is the tandem tag that contains a SBP (45-aa-long tag) and a CBP (26-aa-long tag). Whole-cell lysate from the cells was first bound to streptavidin beads. Unbound proteins were washed twice with the streptavidin-binding buffer, and bound proteins were eluted with the streptavidin elution buffer, which contains 2 mM biotin. The eluate was subsequently bound to calmodulin beads. Unbound proteins were washed twice with calmodulin binding buffer, and bound proteins were eluted with the calmodulin elution buffer. The final eluate containing the protein of interest (Vps41) and the proteins that associate with it were subsequently analyzed by tandem mass spectrometry at the Taplin MS Facility (Harvard Medical School). The result of mass spectrometry is listed in Table S2.

### GST-pulldown and dot-blot assay

For protein expression and purification, bacterial expression vectors encoding for GST or GST-tagged proteins were transformed into *Escherichia coli* BL21 strain. Primary cultures of a transformed single colony were set up for 12 h at 37°C in Luria–Bertani broth containing plasmid vector antibiotic. Secondary cultures were set up in autoinduction media (FORMEDIUM) using 1% primary inoculum and subjected to incubation at 18°C for 30 h. After the incubation period, bacterial cultures were centrifuged at 4,000 rpm for 15 min, washed once with 1× PBS, and resuspended in buffer (20 mM Hepes and 150 mM NaCl, pH 7.4) containing protease inhibitor tablet (Roche) and 1 mM PMSF. Cell lysis was performed by sonication, followed by centrifugation at 12,000 rpm for 15 min at 4°C. The supernatants were incubated with glutathione resin (Gbiosciences) on rotation for 2 h at 4°C to allow binding of GST and GST-tagged proteins, followed by 10 washes with wash buffer (20 mM Hepes, 300 mM NaCl, and 0.5% Triton X-100, pH 7.4).

For pulldown assays, transfected HEK293T cells were lysed in ice-cold TAP lysis buffer, and lysates were incubated with GST-tagged proteins bound to glutathione resin at 4°C for 3 h with rotation. Samples were washed four times with TAP wash buffer, and elution was performed by boiling the samples in Laemmli buffer and loaded onto SDS-PAGE for analysis.

For the dot-blot assay, purified GST and GST-fusion proteins were spotted on nitrocellulose membrane, blocked with 10% skim milk in 0.05% PBS-Tween 20, and washed. The blots were then incubated overnight with purified His-Arl8b and His-Rab7 (in 2% skim milk in 0.05% PBS-Tween 20) at 4°C. The blot was further probed for analysis.

### Yeast two-hybrid and three-hybrid assay

For the yeast two-hybrid assay, plasmids encoding GAL4-AD and GAL4-BD fusion encoding constructs were co-transformed in *Saccharomyces cerevisiae* Gold or AH109 strain (Takara Bio Inc.), streaked on plates lacking leucine and tryptophan and allowed to grow at 30°C for 3 days. The co-transformants were replated on nonselective medium and selective medium to assess interaction. For performing the yeast three-hybrid assay, the *S. cerevisiae* Gold strain was made sensitive to Met by streaking the yeast on an SD-Met plate at least two times before transforming with the desired plasmid.

### DQ Red-BSA trafficking assay

Cells were loaded with DQ Red-BSA (Molecular Probes) at a working concentration of 10 µg/ml in 1% FBS culture medium for 1 h and 6 h at 37°C and 5% CO_2_. In the case of rescue of DQ Red-BSA trafficking, the siRNA-resistant construct of interest was transfected after 50–55 h of siRNA treatment of cells, followed by DQ Red-BSA uptake after 10–12 h of transfection. The cells were fixed in 4% PFA made in PBS (pH 7.4) and analyzed under a confocal microscope. Fold change in total fluorescence intensity of DQ-BSA fluorescence from 1 h to 6 h and the number of DQ Red-BSA spots were quantified using ImageJ software.

### DiI-LDL trafficking assay

Cells were transfected with siRNA of interest for 60–65 h followed by lysosome prelabeling with dextran–Oregon green (Molecular Probes; Thermo Fisher Scientific). In brief, the cells were pulsed with 0.25 mg/ml dextran–Oregon green for 1h followed by a chase for 6 h, the first 3 h of which was done in complete media (10% FBS in DMEM), followed by 3-h starvation in 5% charcoal-stripped FBS (Gibco; Thermo Fisher Scientific) containing DMEM (starvation media). The cells were then pulsed with 20 µg/ml DiI-LDL (Molecular Probes; Thermo Fisher Scientific) for 10 min in starvation media and chased in complete media (DiI-LDL–free medium) for 20 min, 40 min, 1 h, and 1.5 h. Cells were fixed with 4% PFA made in PBS, pH 7.4, at the indicated time points and analyzed by confocal microscopy. The PC of dextran–Oregon green–labeled lysosomes and DiI-LDL was quantified using ImageJ software.

### Autophagy flux assay

Autophagic flux was determined by checking for the rescue of LC3B-II degradation by treating U2OS cells with 100 nM of the V-ATPase inhibitor Baf A1 (for 2 h) either at steady state or with serum starvation in EBSS for 2 h. After treatment, cells were lysed on ice in RIPA buffer supplemented with protease inhibitor. Equal amount of lysates were loaded on SDS-PAGE, transferred to polyvinylidene fluoride membrane, and probed for LC3B-II and α-tubulin. Densitometry analysis of LC3B-II band intensity normalized to α-tubulin intensity was done using ImageJ software.

### Statistical analysis

GraphPad Prism 6 software was used to plot, analyze, and represent the data. Data are presented as means ± SEM. P-values were calculated using two-tailed unpaired Student’s *t* test from three independent biological replicates, and differences were considered significant when P < 0.05. The sample sizes are specified in the figure legends for all of the quantitative data.

### Online supplemental material

Fig. S1 shows that PLEKHM1 directly binds to Arl8b through its N-terminal RUN domain and that its binding to Arl8a is significantly weaker in comparison to Arl8b. In addition, this figure shows that Rab7 regulates membrane localization of PLEKHM1. Fig. S2 shows the clustered endolysosomes and colocalized Rab7/Arl8b endosomes in presence of PLEKHM1 visualized using immuno-EM and super-resolution microscopy. Furthermore, this figure shows colocalization analysis of dextran with LAMP1 in control- and Arl8b-depleted cells. Fig. S3 shows that Vps39’s interaction with PLEKHM1 does not depend on Arl8b, whereas association of other HOPS subunits with PLEKHM1 is Arl8b dependent. Fig. S4 shows the delayed trafficking of endocytic cargo, DiI-LDL, to lysosomes in PLEKHM1-depleted cells. Fig. S5 shows the effect of RILP on lysosome positioning in cells depleted of PLEKHM1. Further, this figure shows that role of conserved arginine residues within the SKIP RUN domain that are required for binding to Arl8b. Video 1 is a time-lapse video showing transient “kiss-and-run” events between GFP-Rab7– and Arl8b-tomato–labeled endosomes. Video 2 shows strong colocalization of GFP-Rab7 and Arl8b-tomato on clustered endolysosomes upon PLEKHM1 overexpression. Video 3 shows lack of association between GFP-Rab7 and Arl8b-tomato compartments upon NΔ300 PLEKHM1 overexpression, a deletion mutant of PLEKHM1 that does not interact with Arl8b. Table S1 details the plasmids used in this study. Table S2 details the mass spectrometry results of eluates from TAP pulldown where TAP-tagged Vps41 was used as bait.

## Supplementary Material

Supplemental Materials (PDF)

Video 1

Video 2

Video 3

## References

[bib1] Balderhaar H.J., and Ungermann C. 2013 CORVET and HOPS tethering complexes: Coordinators of endosome and lysosome fusion. J. Cell Sci. 126:1307–1316. 10.1242/jcs.107805 23645161

[bib2] Burguete A.S., Fenn T.D., Brunger A.T., and Pfeffer S.R. 2008 Rab and Arl GTPase family members cooperate in the localization of the golgin GCC185. Cell. 132:286–298. 10.1016/j.cell.2007.11.048 18243103 PMC2344137

[bib3] Callebaut I., de Gunzburg J., Goud B., and Mornon J.P. 2001 RUN domains: A new family of domains involved in Ras-like GTPase signaling. Trends Biochem. Sci. 26:79–83. 10.1016/S0968-0004(00)01730-8 11166556

[bib4] Costes S.V., Daelemans D., Cho E.H., Dobbin Z., Pavlakis G., and Lockett S. 2004 Automatic and quantitative measurement of protein-protein colocalization in live cells. Biophys. J. 86:3993–4003. 10.1529/biophysj.103.038422 15189895 PMC1304300

[bib5] Dykes S.S., Gray A.L., Coleman D.T., Saxena M., Stephens C.A., Carroll J.L., Pruitt K., and Cardelli J.A. 2016 The Arf-like GTPase Arl8b is essential for three-dimensional invasive growth of prostate cancer in vitro and xenograft formation and growth in vivo. Oncotarget. 7:31037–31052. 27105540 10.18632/oncotarget.8832 PMC5058737

[bib6] Garg S., Sharma M., Ung C., Tuli A., Barral D.C., Hava D.L., Veerapen N., Besra G.S., Hacohen N., and Brenner M.B. 2011 Lysosomal trafficking, antigen presentation, and microbial killing are controlled by the Arf-like GTPase Arl8b. Immunity. 35:182–193. 10.1016/j.immuni.2011.06.009 21802320 PMC3584282

[bib7] Hofmann I., and Munro S. 2006 An N-terminally acetylated Arf-like GTPase is localised to lysosomes and affects their motility. J. Cell Sci. 119:1494–1503. 10.1242/jcs.02958 16537643

[bib8] Jiang P., Nishimura T., Sakamaki Y., Itakura E., Hatta T., Natsume T., and Mizushima N. 2014 The HOPS complex mediates autophagosome-lysosome fusion through interaction with syntaxin 17. Mol. Biol. Cell. 25:1327–1337. 10.1091/mbc.E13-08-0447 24554770 PMC3982997

[bib9] Johnson D.E., Ostrowski P., Jaumouillé V., and Grinstein S. 2016 The position of lysosomes within the cell determines their luminal pH. J. Cell Biol. 212:677–692. 10.1083/jcb.201507112 26975849 PMC4792074

[bib10] Jordens I., Fernandez-Borja M., Marsman M., Dusseljee S., Janssen L., Calafat J., Janssen H., Wubbolts R., and Neefjes J. 2001 The Rab7 effector protein RILP controls lysosomal transport by inducing the recruitment of dynein-dynactin motors. Curr. Biol. 11:1680–1685. 10.1016/S0960-9822(01)00531-0 11696325

[bib11] Kajiho H., Kajiho Y., Frittoli E., Confalonieri S., Bertalot G., Viale G., Di Fiore P.P., Oldani A., Garre M., Beznoussenko G.V., 2016 RAB2A controls MT1-MMP endocytic and E-cadherin polarized Golgi trafficking to promote invasive breast cancer programs. EMBO Rep. 17:1061–1080. 10.15252/embr.201642032 27255086 PMC4931572

[bib12] Khatter D., Raina V.B., Dwivedi D., Sindhwani A., Bahl S., and Sharma M. 2015 a The small GTPase Arl8b regulates assembly of the mammalian HOPS complex on lysosomes. J. Cell Sci. 128:1746–1761. 10.1242/jcs.162651 25908847 PMC4432227

[bib13] Khatter D., Sindhwani A., and Sharma M. 2015 b Arf-like GTPase Arl8: Moving from the periphery to the center of lysosomal biology. Cell. Logist. 5:e1086501 10.1080/21592799.2015.1086501 27057420 PMC4820812

[bib14] Kimura S., Noda T., and Yoshimori T. 2007 Dissection of the autophagosome maturation process by a novel reporter protein, tandem fluorescent-tagged LC3. Autophagy. 3:452–460. 10.4161/auto.4451 17534139

[bib15] Klionsky D.J., Abdelmohsen K., Abe A., Abedin M.J., Abeliovich H., Acevedo Arozena A., Adachi H., Adams C.M., Adams P.D., Adeli K., 2016 Guidelines for the use and interpretation of assays for monitoring autophagy. Autophagy. 12:1–222. 26799652 10.1080/15548627.2015.1100356 PMC4835977

[bib16] Korolchuk V.I., Saiki S., Lichtenberg M., Siddiqi F.H., Roberts E.A., Imarisio S., Jahreiss L., Sarkar S., Futter M., Menzies F.M., 2011 Lysosomal positioning coordinates cellular nutrient responses. Nat. Cell Biol. 13:453–460. 10.1038/ncb2204 21394080 PMC3071334

[bib17] Li X., Rydzewski N., Hider A., Zhang X., Yang J., Wang W., Gao Q., Cheng X., and Xu H. 2016 A molecular mechanism to regulate lysosome motility for lysosome positioning and tubulation. Nat. Cell Biol. 18:404–417. 10.1038/ncb3324 26950892 PMC4871318

[bib18] Lin X., Yang T., Wang S., Wang Z., Yun Y., Sun L., Zhou Y., Xu X., Akazawa C., Hong W., and Wang T. 2014 RILP interacts with HOPS complex via VPS41 subunit to regulate endocytic trafficking. Sci. Rep. 4:7282 10.1038/srep07282 25445562 PMC4250914

[bib19] McEwan D.G., Popovic D., Gubas A., Terawaki S., Suzuki H., Stadel D., Coxon F.P., Miranda de Stegmann D., Bhogaraju S., Maddi K., 2015 a PLEKHM1 regulates autophagosome-lysosome fusion through HOPS complex and LC3/GABARAP proteins. Mol. Cell. 57:39–54. 10.1016/j.molcel.2014.11.006 25498145

[bib20] McEwan D.G., Richter B., Claudi B., Wigge C., Wild P., Farhan H., McGourty K., Coxon F.P., Franz-Wachtel M., Perdu B., 2015 b PLEKHM1 regulates *Salmonella*-containing vacuole biogenesis and infection. Cell Host Microbe. 17:58–71. 10.1016/j.chom.2014.11.011 25500191

[bib21] Michelet X., Garg S., Wolf B.J., Tuli A., Ricciardi-Castagnoli P., and Brenner M.B. 2015 MHC class II presentation is controlled by the lysosomal small GTPase, Arl8b. J. Immunol. 194:2079–2088. 10.4049/jimmunol.1401072 25637027

[bib22] Mrakovic A., Kay J.G., Furuya W., Brumell J.H., and Botelho R.J. 2012 Rab7 and Arl8 GTPases are necessary for lysosome tubulation in macrophages. Traffic. 13:1667–1679. 10.1111/tra.12003 22909026

[bib23] Pankiv S., Alemu E.A., Brech A., Bruun J.A., Lamark T., Overvatn A., Bjørkøy G., and Johansen T. 2010 FYCO1 is a Rab7 effector that binds to LC3 and PI3P to mediate microtubule plus end-directed vesicle transport. J. Cell Biol. 188:253–269. 10.1083/jcb.200907015 20100911 PMC2812517

[bib24] Pu J., Guardia C.M., Keren-Kaplan T., and Bonifacino J.S. 2016 Mechanisms and functions of lysosome positioning. J. Cell Sci. 129:4329–4339. 10.1242/jcs.196287 27799357 PMC5201012

[bib25] Reinecke J.B., Katafiasz D., Naslavsky N., and Caplan S. 2015 Novel functions for the endocytic regulatory proteins MICAL-L1 and EHD1 in mitosis. Traffic. 16:48–67. 10.1111/tra.12234 25287187 PMC4275409

[bib26] Rocha N., Kuijl C., van der Kant R., Janssen L., Houben D., Janssen H., Zwart W., and Neefjes J. 2009 Cholesterol sensor ORP1L contacts the ER protein VAP to control Rab7-RILP-p150 Glued and late endosome positioning. J. Cell Biol. 185:1209–1225. 10.1083/jcb.200811005 19564404 PMC2712958

[bib27] Rosa-Ferreira C., and Munro S. 2011 Arl8 and SKIP act together to link lysosomes to kinesin-1. Dev. Cell. 21:1171–1178. 10.1016/j.devcel.2011.10.007 22172677 PMC3240744

[bib28] Schiefermeier N., Scheffler J.M., de Araujo M.E., Stasyk T., Yordanov T., Ebner H.L., Offterdinger M., Munck S., Hess M.W., Wickström S.A., 2014 The late endosomal p14-MP1 (LAMTOR2/3) complex regulates focal adhesion dynamics during cell migration. J. Cell Biol. 205:525–540. 10.1083/jcb.201310043 24841562 PMC4033770

[bib29] Shi A., and Grant B.D. 2013 Interactions between Rab and Arf GTPases regulate endosomal phosphatidylinositol-4,5-bisphosphate during endocytic recycling. Small GTPases. 4:106–109. 10.4161/sgtp.23477 23392104 PMC3747250

[bib30] Tabata K., Matsunaga K., Sakane A., Sasaki T., Noda T., and Yoshimori T. 2010 Rubicon and PLEKHM1 negatively regulate the endocytic/autophagic pathway via a novel Rab7-binding domain. Mol. Biol. Cell. 21:4162–4172. 10.1091/mbc.E10-06-0495 20943950 PMC2993745

[bib31] Tuli A., Thiery J., James A.M., Michelet X., Sharma M., Garg S., Sanborn K.B., Orange J.S., Lieberman J., and Brenner M.B. 2013 Arf-like GTPase Arl8b regulates lytic granule polarization and natural killer cell-mediated cytotoxicity. Mol. Biol. Cell. 24:3721–3735. 10.1091/mbc.E13-05-0259 24088571 PMC3842998

[bib32] van der Kant R., Fish A., Janssen L., Janssen H., Krom S., Ho N., Brummelkamp T., Carette J., Rocha N., and Neefjes J. 2013 Late endosomal transport and tethering are coupled processes controlled by RILP and the cholesterol sensor ORP1L. J. Cell Sci. 126:3462–3474. 10.1242/jcs.129270 23729732

[bib33] Van Wesenbeeck L., Odgren P.R., Coxon F.P., Frattini A., Moens P., Perdu B., MacKay C.A., Van Hul E., Timmermans J.P., Vanhoenacker F., 2007 Involvement of PLEKHM1 in osteoclastic vesicular transport and osteopetrosis in incisors absent rats and humans. J. Clin. Invest. 117:919–930. 10.1172/JCI30328 17404618 PMC1838941

[bib34] Wang T., Ming Z., Xiaochun W., and Hong W. 2011 Rab7: Role of its protein interaction cascades in endo-lysosomal traffic. Cell. Signal. 23:516–521. 10.1016/j.cellsig.2010.09.012 20851765

[bib35] Wijdeven R.H., Janssen H., Nahidiazar L., Janssen L., Jalink K., Berlin I., and Neefjes J. 2016 Cholesterol and ORP1L-mediated ER contact sites control autophagosome transport and fusion with the endocytic pathway. Nat. Commun. 7:11808 10.1038/ncomms11808 27283760 PMC4906411

[bib36] Witwicka H., Jia H., Kutikov A., Reyes-Gutierrez P., Li X., and Odgren P.R. 2015 TRAFD1 (FLN29) interacts with Plekhm1 and regulates osteoclast acidification and resorption. PLoS One. 10:e0127537 10.1371/journal.pone.0127537 25992615 PMC4438057

