# Supplementary material for: The Rab7 effector PLEKHM1 binds Arl8b to promote cargo traffic to lysosomes
Source: J Cell Biol. 2017 Apr 3;216(4):1051–70. doi: 10.1083/jcb.201607085 (PMC5379943; doi:10.1083/jcb.201607085)
Supplement: Supplemental Materials (PDF) [file JCB_201607085_sm.pdf]

Marwaha et al., https://doi.org/10.1083/jcb.201607085

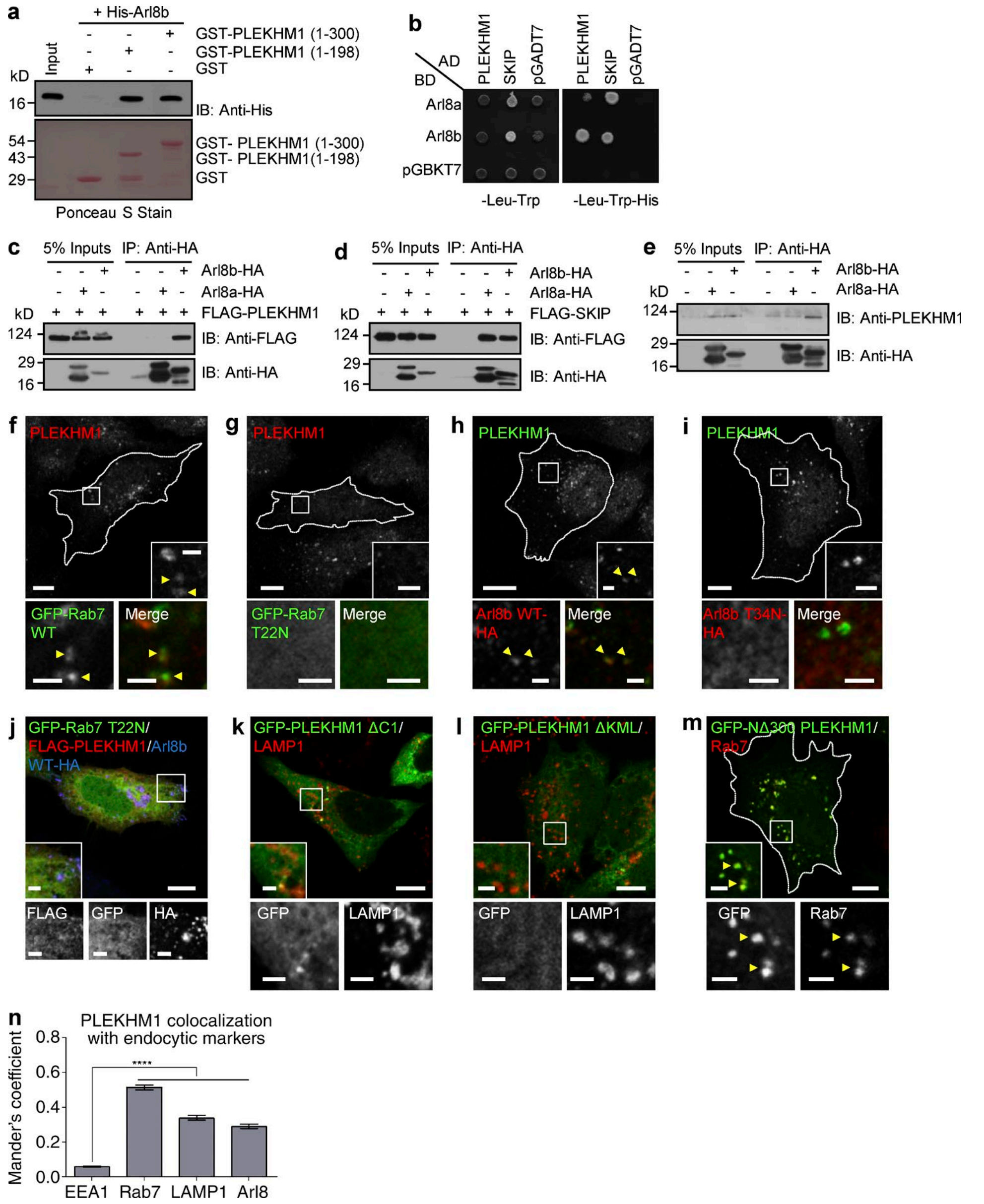

Figure S1. **PLEKHM1 interacts weakly with Arl8a and is recruited to membranes by Rab7.** (a) Immunoblot (IB) showing direct binding of His-Arl8b incubated with GST alone, GST-PLEKHM1 (1–198), and GST-PLEKHM1 (1–300). (b) Interaction of PLEKHM1 with Arl8a was tested using the yeast two-hybrid assay. Cotransformants expressing the indicated proteins were spotted on nonselective medium (-Leu-Trp) to check viability and on selective medium (-Leu-Trp-His) to assess the interaction. (c and d) HEK293T cell lysates expressing FLAG-PLEKHM1 (c) or FLAG-SKIP (d) alone and coexpressed with Arl8a-HA or with Arl8b-HA were immunoprecipitated (IP) with anti-HA Abs-resin and immunoblotted with the indicated antibodies. (e) Western blot of HEK293T lysates expressing either Arl8a-HA or Arl8b-HA IP with anti–HA antibody resin and probed with anti–PLEKHM1 antibody. (f and g) Representative confocal micrographs of HeLa cells transfected with either GFP-Rab7 WT or GFP-Rab7 T22N and immunostained for PLEKHM1. Colocalized pixels are marked by arrowheads in the insets. (h and i) Representative confocal images of HeLa cells transfected with either Arl8b WT-HA or Arl8b T34N-HA and immunostained for PLEKHM1. (j) Representative confocal micrograph of HeLa cells transfected with FLAG-PLEKHM1, Arl8b-HA, and GFP-Rab7 T22N and immunostained with anti–FLAG and anti–HA antibodies. (k and l) Representative confocal micrographs of HeLa cells transfected with Rab7-binding–defective mutants of PLEKHM1 and immunostained for LAMP1. (m) Representative confocal micrograph of HeLa cells transfected with GFP-NΔ300 PLEKHM1 and stained for Rab7. Colocalized pixels are marked by arrowheads in the insets. (n) Colocalization of PLEKHM1 with different endocytic markers was assessed by measuring the Mander's coefficient (n = 3; 25–30 cells analyzed per experiment). Data represent mean ± SEM (****, $P < 0.0001$; Student's t test). Bars: (main) 10 µm; (insets) 2 µm.

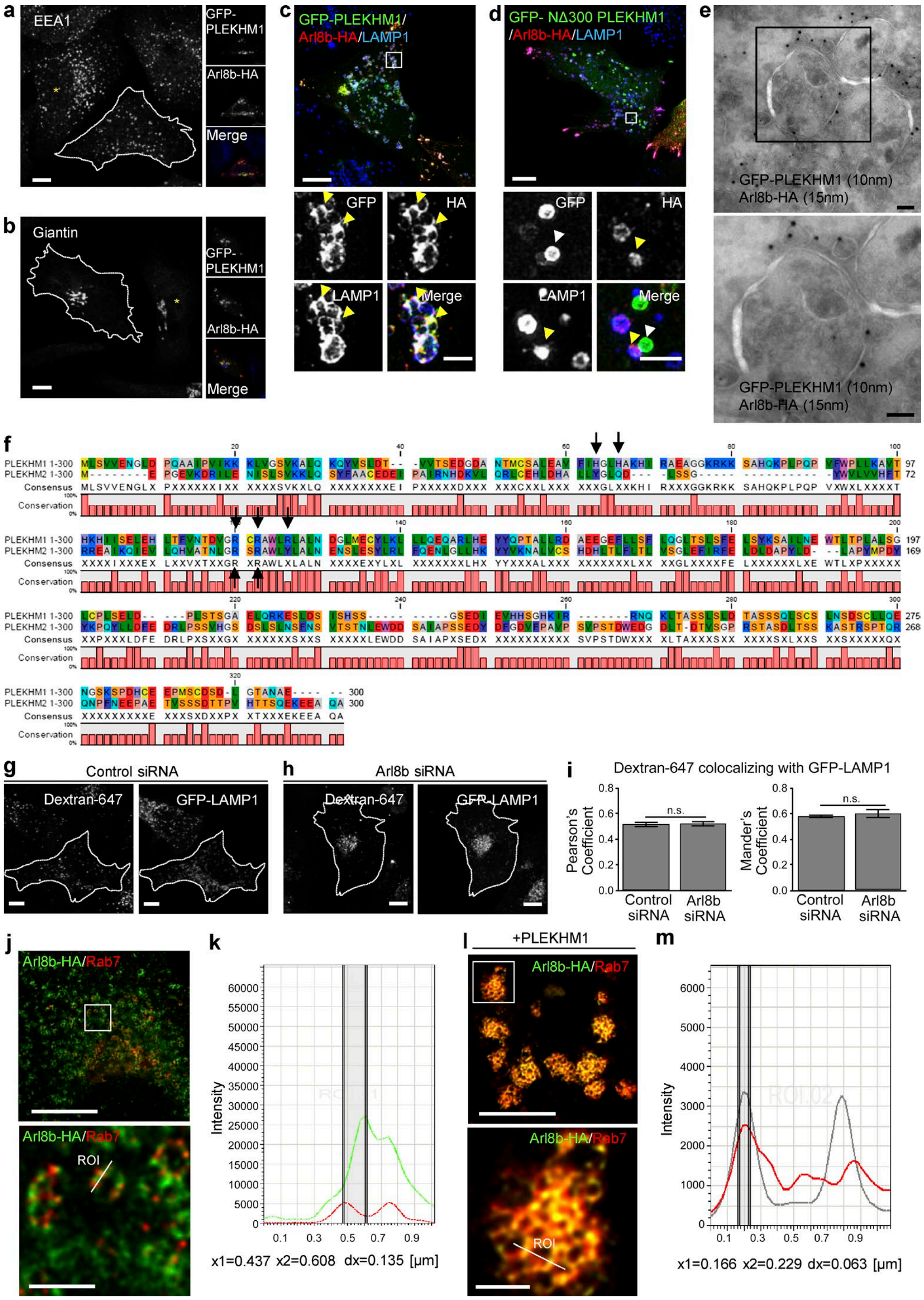

Figure S2. **PLEKHM1 colocalizes with Arl8b on lysosomes and promotes clustering of Rab7-positive LEs and Arl8b-positive lysosomes.** (a and b) Representative confocal micrograph of HeLa cells cotransfected with Arl8b-HA and GFP-PLEKHM1 and stained for the early endosomal marker EEA1 or the Golgi marker Giantin (asterisk marks untransfected cells). (c and d) SIM image of HeLa cells cotransfected with Arl8b-HA and GFP-PLEKHM1 or GFP-NΔ300 PLEKHM1 and stained for lysosomes using anti–LAMP1 antibodies. In the insets, yellow arrowheads indicate colocalized pixels and white arrowheads denote NΔ300 PLEKHM1-positive vesicles. (e) HeLa cells cotransfected with GFP-PLEKHM1 and Arl8b-HA were fixed, labeled, and analyzed by cryo–immunogold EM. Boxed area is magnified below. Bar, 100 nm. (f) Sequence alignment showing 1- to 300-aa fragments of PLEKHM1 and PLEKHM2/SKIP. Black arrows mark the basic/positively charged residues of PLEKHM1 and PLEKHM2/SKIP mutated in the respective RUN domains of the two proteins used in this study. (g–i) Control- or Arl8b-siRNA–treated HeLa cells were incubated overnight with dextran-647 followed by transfection of GFP-LAMP1, and their colocalization was analyzed by confocal microscopy. Colocalization was also quantified by measuring PC and MC (n = 3; 30 cells analyzed per experiment for each treatment). (j) STED image of HeLa cells expressing Arl8b-HA (pseudo color green) and immunostained for Rab7. (k) Intensity profile of ROI from j. Note the separation (dx) between the Arl8b-HA and Rab7 intensity peak is 0.135 µm. In the graph, green line indicates Arl8b-HA signal and red line indicates Rab7 signal. (l) STED image of HeLa cells cotransfected with Arl8b-HA (pseudo color green) and FLAG-PLEKHM1 (not stained) and immunostained for Rab7. (m) Intensity profile of ROI from k. Note the separation (dx) between the Arl8b-HA and Rab7 intensity peak is reduced to 0.063 µm. In the graph, gray line indicates Arl8b-HA signal and red line indicates Rab7 signal. All the graphs represent mean ± SEM (n.s., not significant; Student's t test). Bars: (main) 10 µm; (insets) 2 µm.

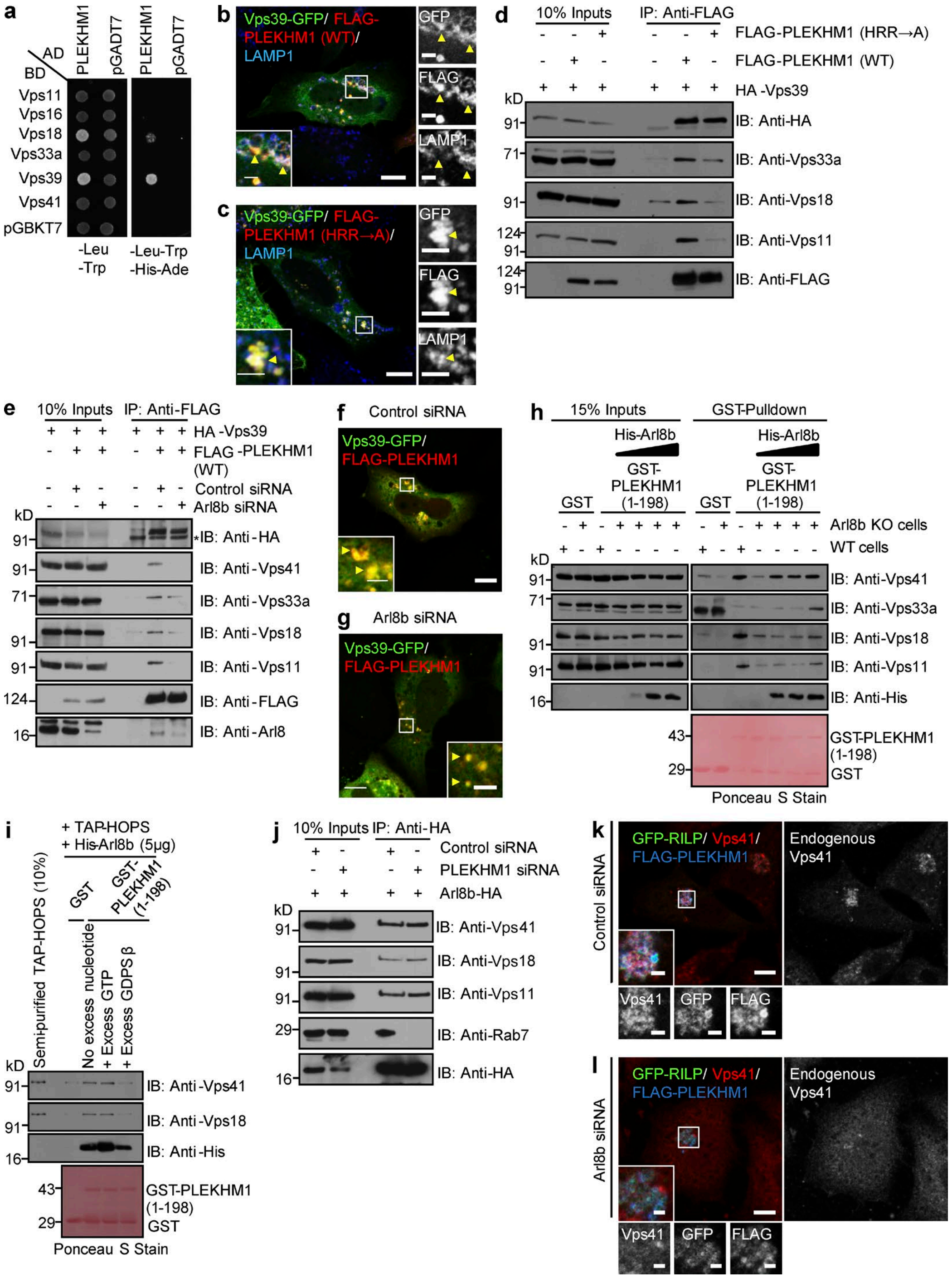

Figure S3. **PLEKHM1 directly interacts with Vps39 but requires Arl8b for its interaction with other subunits of the HOPS complex.** (a) Interaction of individual HOPS complex subunits with PLEKHM1 was tested using the yeast two-hybrid assay. The cotransformants were spotted on nonselective medium (-Leu-Trp) to check for viability and on selective medium (-Leu-Trp-His-Ade) to detect interaction. (b and c) Representative confocal micrographs of HeLa cells coexpressing Vps39-GFP with FLAG-PLEKHM1 or FLAG-PLEKHM1 (HRR→A) and immunostained with anti–LAMP1 antibodies. Colocalized pixels are marked by arrowheads in the insets. (d) Lysates from HEK293T cells coexpressing HA-Vps39 along with FLAG-PLEKHM1 or FLAG-PLEKHM1 (HRR→A) were immunoprecipitated (IP) using anti–FLAG antibody resin and immunoblotted (IB) with the indicated antibodies against the different HOPS subunits. (e) HEK293T cells treated with control- or Arl8b-siRNA and coexpressing HA-Vps39 and FLAG-PLEKHM1 were immunoprecipitated with anti–FLAG antibody resin and immunoblotted with the indicated antibodies. The asterisk indicate nonspecific signal observed in all the lanes. (f and g) Representative confocal images of HeLa cells treated with either control- or Arl8b-siRNA and cotransfected with Vps39-GFP and FLAG-PLEKHM1. Colocalized pixels are marked by arrowheads in the insets. (h) Western blot of GST-pulldown assay using GST alone or GST-PLEKHM1 (1–198) as a bait incubated with lysates from WT- or Arl8b KO-HeLa cells and increasing concentration of His-Arl8b protein and immunoblotted with the indicated antibodies. (i) Western blot analysis with indicated antibodies of semipurified TAP–HOPS complex isolated from HeLa cells and incubated with GST alone or GST-PLEKHM1 (1–198), His-Arl8b, and excess GTP or GDP. (j) Western blot of control- or PLEKHM1-siRNA–treated HEK293T cell lysates expressing Arl8b-HA and immunoprecipitated with anti–HA antibody and immunoblotted with the indicated antibodies. (k and l) Representative confocal micrographs of HeLa cells treated with either control- or Arl8b-siRNA and cotransfected with GFP-RILP and FLAG-PLEKHM1 and immunostained for Vps41. Different channels are shown in the insets. Bars: (main) 10 µm; (insets) 2 µm.

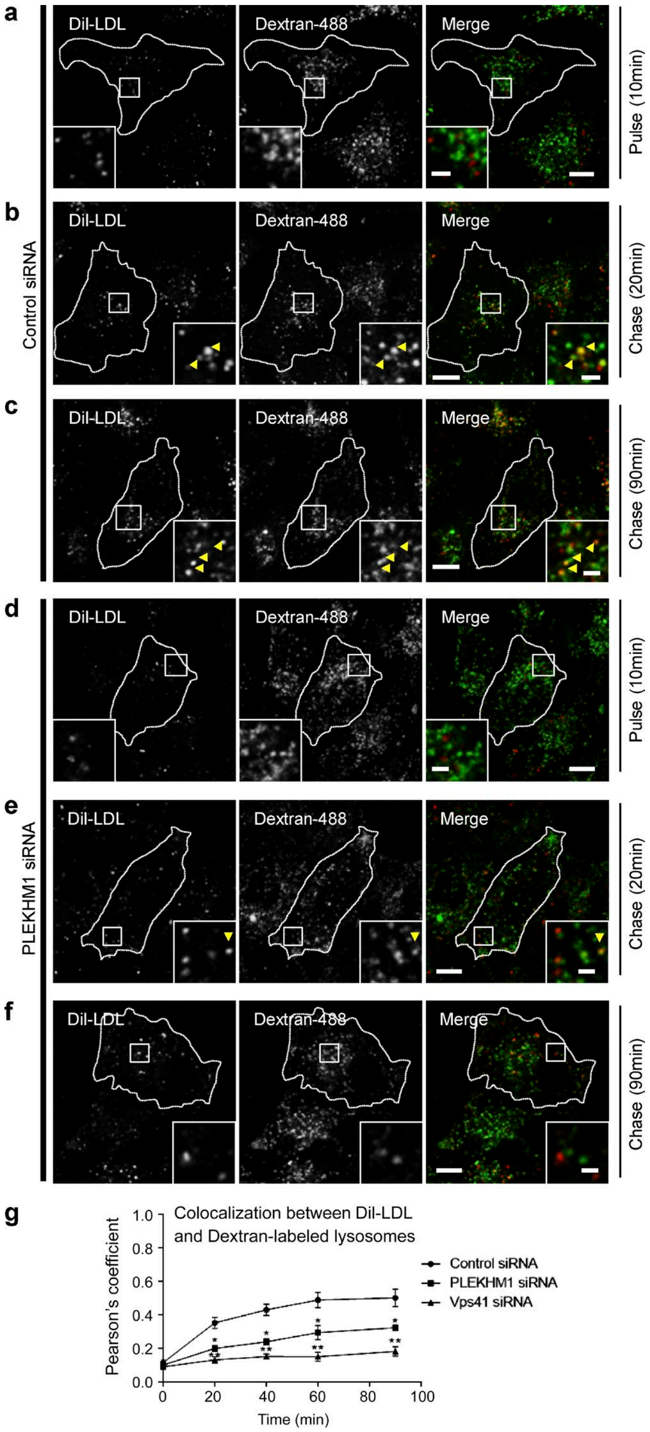

Figure S4. **PLEKHM1 depletion delays DiI-LDL trafficking to lysosomes.** (a–f) Lysosomes of HeLa cells treated with control- or PLEKHM1-siRNA were prelabeled with dextran-488 followed by starvation in media containing 5% charcoal-stripped FBS, which leads to LDL-R accumulation on the membrane. Cells were then pulsed with DiI-LDL for 10 min and chase in complete media for indicated time points. Shown are the representative confocal micrographs of LDL trafficking in control- and PLEKHM1-depleted cells. Arrowheads indicate colocalized pixels. (g) Colocalization between DiI-LDL– and dextran-labeled lysosomes for indicated time points in control-, PLEKHM1-, or Vps41-siRNA–treated HeLa cells was quantified by measuring PC (n = 3; 30 cells analyzed per time point for each treatment). Data represent mean ± SEM (*, $P < 0.05$; **, $P < 0.01$; Student's t test). Bars: (main) 10 µm; (insets) 2 µm.

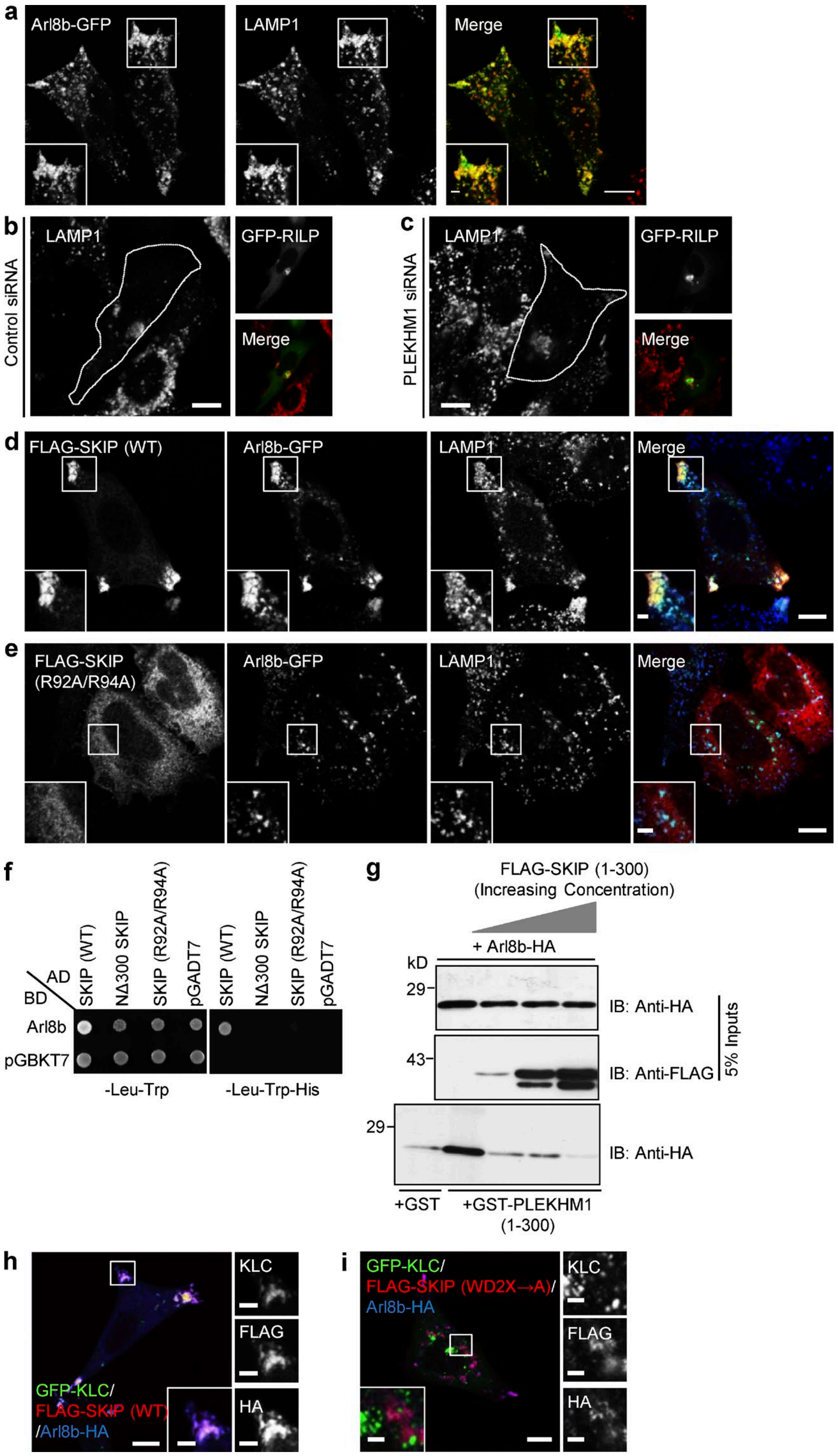

Figure S5. **Conserved basic residues within the RUN domain of SKIP are required for its interaction with Arl8b.** (a) Representative confocal image of HeLa cells transfected with Arl8b-GFP and stained for lysosomes using anti–LAMP1 antibodies. Colocalization between Arl8b and LAMP1 compartment can be seen in the inset. (b and c) Representative confocal image of HeLa cells treated with control- or PLEKHM1-siRNA and transfected with GFP-RILP and immunostained with anti–LAMP1 antibodies. (d and e) Representative confocal images of HeLa cells expressing Arl8b-GFP along with FLAG-SKIP (WT) or FLAG-SKIP (R92A/R94A) mutant and stained with anti–LAMP1 antibodies. (f) Interaction of SKIP (WT), NΔ300 SKIP, and SKIP (R92A/R94A) mutant with Arl8b was tested in a yeast two-hybrid assay. (g) Lysates from HEK293T cells cotransfected with Arl8b-HA and vector or increasing amounts of FLAG-SKIP (1–300) were incubated with GST or GST-PLEKHM1 (1–300) and analyzed by Western blotting using the indicated antibodies. (h and i) Representative confocal micrographs of HeLa cells transfected with Arl8-HA, GFP-KLC2 with FLAG-SKIP (WT), or FLAG-SKIP (WD2X→A) mutant. Bars: (main) 10 µm; (insets) 2 µm.

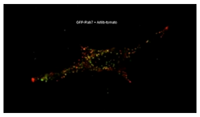

Video 1. **Transient kiss-and-run interactions between Rab7$^+$- and Arl8b$^+$-endosomes.** Live-cell imaging of HeLa cells coexpressing GFP-Rab7 and Arl8b-tomato with every image captured at an interval of 2.32 s (total number of frames captured = 150). Movie is shown at seven frames per second.

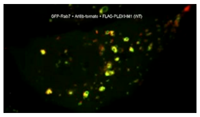

Video 2. **Rab7 and Arl8b remain highly colocalized on the clustered and enlarged endolysosomes upon expression of PLEKHM1.** Live-cell imaging was performed on HeLa cells coexpressing GFP-Rab7, Arl8b-tomato, and FLAG-PLEKHM1 (WT; unstained) with every image captured at an interval of 2.64 s (total number of frames captured = 150). Movie is shown at seven frames per second.

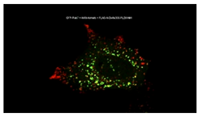

Video 3. **Reduced colocalization between Rab7$^+$ and Arl8b$^+$ endosomes upon expression of Arl8b-binding–defective mutant of PLEKHM1.** Live-cell imaging was performed on HeLa cells coexpressing GFP-Rab7, Arl8b-tomato, and FLAG-NΔ300 PLEKHM1 (unstained) with every image captured at an interval of 2.64 s (total number of frames captured = 150). Movie is shown at seven frames per second.

Table S1. **List of molecular constructs used in this study**

Plasmid name	Description	Source
**Yeast two-hybrid constructs**		
pGADT7 vector	GAL4-activation domain yeast two-hybrid vector	Takara Bio Inc.
pGADT7-PLEKHM1 (WT)	Full-length human PLEKHM1 (1–1,056 aa) cloned into the pGADT7 vector	This study
pGADT7-NΔ198 PLEKHM1	Human PLEKHM1 (199–1,056 aa) cloned into the pGADT7 vector	This study
pGADT7-NΔ300 PLEKHM1	Human PLEKHM1 (301–1,056 aa) cloned into the pGADT7 vector	This study
pGADT7-PLEKHM1 (H60A)	Human PLEKHM1 with point mutation at amino acid position 60 changing H with A; cloned into the pGADT7 vector	This study
pGADT7-PLEKHM1 (H63A)	Human PLEKHM1 with point mutation at amino acid position 63 changing H with A; cloned into the pGADT7 vector	This study
pGADT7-PLEKHM1 (RR→A)	Human PLEKHM1 with point mutations at amino acid positions 117 and 119 changing both R with A; cloned into the pGADT7 vector	This study
pGADT7-PLEKHM1 (R123A)	Human PLEKHM1 with point mutation at amino acid position 123 changing R with A; cloned into the pGADT7 vector	This study
pGADT7-PLEKHM1 (HRR→A)	Human PLEKHM1 with point mutations at amino acid positions 60, 117 and 119 changing H with A and both R with A, respectively; cloned into the pGADT7 vector	This study
pGADT7-SKIP (WT)	Full-length human SKIP (1–1,019 aa) cloned into the pGADT7 vector	Khatter et al., 2015
pGADT7-NΔ300 SKIP	Human SKIP (301–772 aa) cloned into the pGADT7 vector	This study
pGADT7-SKIP (R92A/R94A)	Human SKIP with point mutations at amino acid positions 92 and 94 changing both R with A; cloned into the pGADT7 vector	This study
pGBKT7 vector	GAL4-DNA binding domain yeast two-hybrid vector	Takara Bio Inc.
pGBKT7-Arl8a	Human Arl8a (lacking first 17 aa) cloned into the pGBKT7 vector	This study
pGBKT7-Arl8b (WT)	Human Arl8b (lacking first 17 aa) cloned into the pGBKT7 vector	This study
pGBKT7-Arl8b (Q75L)	Human Arl8b (lacking first 17 aa) with Q75L point mutation cloned into the pGBKT7 vector	This study
pGBKT7-Arl8b (T34N)	Human Arl8b (lacking first 17 aa) with T34N point mutation cloned into the pGBKT7 vector	This study
pGBDC1-Rab7	Human Rab7 cloned into the pGBDC1 vector	Gift from T. Yoshimori
pGBKT7-LC3B	Human LC3B cloned into the pGBKT7 vector	This study
pGBKT7-Vps11	Full-length human Vps11 cloned into the pGBKT7 vector	Khatter et al., 2015
pGBKT7-Vps16	Full-length human Vps16 cloned into the pGBKT7 vector	Khatter et al., 2015
pGBKT7-Vps18	Full-length human Vps18 cloned into the pGBKT7 vector	Khatter et al., 2015
pGBKT7-Vps33a	Full-length human Vps33a cloned into the pGBKT7 vector	Khatter et al., 2015
pGBKT7-Vps39	Full-length human Vps39 cloned into the pGBKT7 vector	Khatter et al., 2015
pGBKT7-Vps41	Full-length human Vps41 cloned into the pGBKT7 vector	Khatter et al., 2015
**Yeast three-hybrid constructs**		
pBridge vector	Yeast three-hybrid vector	Takara Bio Inc.
pBridge-Arl8b	Arl8b (lacking first 17 aa) cloned into the MCS-I of the pBridge vector	This study
pBridge-Arl8b/PLEKHM1 (WT)	Arl8b (lacking first 17 aa) cloned into the MCS-I and full-length PLEKHM1 cloned into the MCS-II of the pBridge vector	This study
pBridge-Arl8b/PLEKHM1 (HRR→A)	Arl8b (lacking first 17 aa) cloned into the MCS-I and PLEKHM1 with point mutations at amino acid positions 60, 117 and 119 changing H with A and both R with A, respectively, cloned into the MCS-II of the pBridge vector	This study
**Mammalian expression constructs**		
pcDNA3.1(–)	Mammalian expression vector	Invitrogen
pcDNA3.1(–)-FLAG- PLEKHM1 (WT)	N-terminal FLAG-tagged full-length human PLEKHM1 (1–1,056 aa) cloned into the pcDNA3.1(–) vector	This study
pcDNA3.1(–)-FLAG-PLEKHM1 (H60A)	N-terminal FLAG-tagged human PLEKHM1 with point mutation at amino acid position 60 changing H with A; cloned into the pcDNA3.1(–) vector	This study
pcDNA3.1(–)-FLAG-PLEKHM1 (H63A)	N-terminal FLAG-tagged human PLEKHM1 with point mutation at amino acid position 63 changing H with A; cloned into the pcDNA3.1(–) vector	This study
pcDNA3.1(–)-FLAG-PLEKHM1 (RR→A)	N-terminal FLAG-tagged human PLEKHM1 with point mutations at amino acid positions 117 and 119 changing both R with A; cloned into the pcDNA3.1(–) vector	This study
pcDNA3.1(–)-FLAG-PLEKHM1 (HRR→A)	N-terminal FLAG-tagged human PLEKHM1 with point mutations at amino acid positions 60, 117 and 119 changing H with A and both R with A, respectively; cloned into the pcDNA3.1(–) vector	This study
pcDNA3.1(–)-FLAG-NΔ198 PLEKHM1	N-terminal FLAG-tagged human PLEKHM1 (199–1,056 aa) cloned into the pcDNA3.1(–) vector	This study
pcDNA3.1(–)-FLAG-NΔ300 PLEKHM1	N-terminal FLAG-tagged human PLEKHM1 (301–1,056 aa) cloned into the pcDNA3.1(–) vector	This study
pcDNA3.1(–)-FLAG-PLEKHM1 (WT) siRNA resistant	N-terminal FLAG-tagged full-length human PLEKHM1 (1–1,056 aa) rescue construct against PLEKHM1 siRNA #2 cloned into the pcDNA3.1(–) vector	This study

Table S1. **List of molecular constructs used in this study** *(Continued)*

Plasmid name	Description	Source
pcDNA3.1(–)-FLAG-PLEKHM1 (HRR→A) siRNA resistant	N-terminal FLAG-tagged full-length human PLEKHM1 (1–1,056 aa) rescue construct against PLEKHM1 siRNA #2 with H60A/R117A/R119A point mutations cloned into the pcDNA3.1(–) vector	This study
pEGFPC1-PLEKHM1 (WT)	N-terminal GFP-tagged full-length human PLEKHM1 (1–1,056 aa) cloned into the pEGFP-C1 vector	Gift from T. Yoshimori
pEGFPC1-PLEKHM1 (HRR→A)	N-terminal GFP-tagged full-length human PLEKHM1 (1–1,056 aa) with point mutations at amino acid positions 60, 117 and 119 changing H with A and both R with A, respectively; cloned into the pEGFP-C1 vector	This study
pEGFPC1-NΔ198 PLEKHM1	N-terminal GFP-tagged human PLEKHM1 (199-1056 aa) cloned into the pEGFP-C1 vector	This study
pEGFPC1-NΔ300 PLEKHM1	N-terminal GFP-tagged human PLEKHM1 (301–1,056 aa) cloned into the pEGFP-C1 vector	This study
pEGFPC1-PLEKHM1 ΔC1	N-terminal GFP-tagged human PLEKHM1 (1–895 aa) cloned into the pEGFP-C1 vector	This study
pEGFPC1-PLEKHM1 ΔKML	N-terminal GFP-tagged human PLEKHM1 (lacking 720–722 aa) cloned into the pEGFP-C1 vector	This study
pEGFPC1-PLEKHM1 (WT) siRNA resistant	N-terminal GFP-tagged full-length human PLEKHM1 (1–1,056 aa) rescue construct against PLEKHM1 siRNA #2 cloned into the pEGFPC1 vector	This study
pEGFPC1-PLEKHM1 (HRR→A) siRNA resistant	N-terminal GFP-tagged full-length human PLEKHM1 (1–1,056 aa) rescue construct against PLEKHM1 siRNA #2 with H60A/R117A/R119A point mutations cloned into the pEGFPC1 vector	This study
pcDNA3.1(–)-FLAG-SKIP	N-terminal FLAG-tagged full-length human SKIP (1–1,019 aa) cloned into the pcDNA3.1(–) vector	Khatter et al., 2015
pcDNA3.1(–)-FLAG-SKIP (1-300) only	N-terminal FLAG-tagged human SKIP (1–300 aa) cloned into the pcDNA3.1(–) vector	Khatter et al., 2015
pcDNA3.1(–)-FLAG-SKIP (R92A/R94A)	N-terminal FLAG-tagged human SKIP with point mutations at amino acid positions 92 and 94 changing both R with A; cloned into the pcDNA3.1(–) vector	This study
pcDNA3.1(–)-FLAG-SKIP (WD 2X→A)	N-terminal FLAG-tagged human SKIP with point mutations W207A/D208A/W236A/E237A; cloned into the pcDNA3.1(–) vector	This study
pEGFPC1-SKIP (WT)	N-terminal GFP-tagged full-length human SKIP (1–1,019 aa) cloned into the pEGFPC1 vector	This study
pcDNA3.1(–)-Arl8a (WT)-HA	Full-length human Arl8a with C-terminal HA tag cloned into the pcDNA3.1(–) vector	This study
pcDNA3.1(–)-Arl8b (WT)-HA	Full-length human Arl8b with C-terminal HA tag cloned into the pcDNA3.1(–) vector	Khatter et al., 2015
pcDNA3.1(–)-Arl8b (Q75L)-HA	Full-length human Arl8b Q75L with C-terminal HA tag cloned into the pcDNA3.1(–) vector	Khatter et al., 2015
pcDNA3.1(–)-Arl8b (T34N)-HA	Full-length human Arl8b T34N with C-terminal HA tag cloned into the pcDNA3.1(–) vector	Khatter et al., 2015
ptdTomato-N1-Arl8b	Full-length human Arl8b cloned into the ptdTomato-N1 vector	Khatter et al., 2015
ptdTomato-N1-Arl8b rescue	Full-length human Arl8b siRNA rescue (against siRNA #1) construct cloned into the ptdTomato-N1 vector	Khatter et al., 2015
pcDNA3.1(+)-Mouse Arl8b-GFP	Full-length mouse Arl8b with C-terminal GFP tag cloned into the pcDNA3.1(+) vector	Garg et al., 2011
pEBB-HA-Rab7	Full-length human Rab7 with N-terminal HA tag cloned into the pEBB vector	Gift from J. Kinchen
pEGFPC1-Rab7	N-terminal GFP-tagged full-length canine Rab7 cloned into the pEGFPC1 vector	Gift from S. Caplan
pEGFPC1-Rab7 Q75L	N-terminal GFP-tagged full-length canine Rab7 Q75L cloned into the pEGFPC1 vector	Gift from S. Caplan
pEGFPC1-Rab7 T22N	N-terminal GFP-tagged full-length canine Rab7 T22N cloned into the pEGFPC1 vector	Gift from S. Caplan
pcDNA3.1(–)-HA-Vps41	Full-length human Vps41 with N-terminal HA tag cloned into the pcDNA3.1(–) vector	Khatter et al., 2015
pEGFPC1-Vps41	N-terminal GFP-tagged full-length human Vps41 cloned into the pEGFPC1 vector	This study
N-TAP-Vps41-pCDH-CMV-MCS-EF1-Hygro	N-terminal TAP-tagged full-length human Vps41 cloned into the pCDH-CMV-MCS-EF1-Hygro vector	This study
pcDNA3.1(-)-HA-Vps39	Full-length human Vps39 with N-terminal HA tag cloned into the pcDNA3.1(–) vector	Khatter et al., 2015
pEGFPC1-Vps39	N-terminal GFP-tagged mouse Vps39 cloned into the pEGFPC1 vector	Gift from R. Piper
pEGFPC1-RILP	N-terminal GFP-tagged RILP cloned into the pEGFPC1 vector	Gift from J. Neefjes
pEGFPC1-KLC2	N-terminal GFP-tagged KLC2 cloned into the pEGFPC1 vector	Gift from M. Way
ptf-LC3B	Rat LC3B fused to mRFP and EGFP cloned into pEGFPC1	Gift from T. Yoshimori
pEGFPC1-Lamp1	N-terminal GFP-tagged Lamp1 cloned into the pEGFPC1 vector	Gift from S. Caplan
**Bacterial expression constructs**		
pGEX6P2-PLEKHM1 (1–198)	Human PLEKHM1 (1–198 aa) cloned into the pGEX6P2 vector	This study
pGEX6P2-PLEKHM1 (1–300)	Human PLEKHM1 (1–300 aa) cloned into the pGEX6P2 vector	This study
pET15b(+)-PLEKHM1 (1–300)	Human PLEKHM1 (1–300 aa) with N-terminal His tag cloned into the pET15b(+) vector	This study
pGEX6P2-PLEKHM1 H60A (1–300)	Human PLEKHM1 (1–300 aa) with point mutation at amino acid position 60 changing H with A; cloned into the pGEX6P2 vector	This study
pGEX6P2-PLEKHM1 H63A (1–300)	Human PLEKHM1 (1–300 aa) with point mutation at amino acid position 63 changing H with A; cloned into the pGEX6P2 vector	This study
pGEX6P2-PLEKHM1 RR→A (1–300)	Human PLEKHM1 (1–300 aa) with point mutations at amino acid positions 117 and 119 changing both R with A; cloned into the pGEX6P2 vector	This study

Table S1. **List of molecular constructs used in this study** *(Continued)*

Plasmid name	Description	Source
pGEX6P2-PLEKHM1 HRR→A (1–300)	Human PLEKHM1 (1–300 aa) with point mutations at amino acid positions 60, 117 and 119 changing H with A and both R with A, respectively; cloned into the pGEX6P2 vector	This study
pMAL-C2X-SKIP (1-300)	Human SKIP (1–300 aa) with N-terminal MBP tag cloned into the pMAL-C2X vector	This study
pet15b(+)-Arl8b	Full-length human Arl8b with N-terminal His tag cloned into the pet15b(+) vector	Gift from M. Brenner
pRSF-His-Rab7	Rab7 with N-terminal His tag cloned into the pRSF vector	Gift from A. Spang
pGEX6P2-Rab7	Rab7 cloned into the pGEX6P2 vector	This study

The authors like to thank S. Caplan (University of Nebraska Medical Center, Omaha, NE), M. Brenner (Harvard Medical School, Boson, MA), T. Yoshimori (Osaka University, Osaka, Japan), J. Kinchen (University of Virginia, Charlottesville, VA), J. Neefjes (The Netherlands Cancer Institute, Amsterdam, Netherlands), M. Way (London Research Institute, London, England, UK), A. Spang (University of Basel, Basel, Switzerland), and R. Piper (University of Iowa, Iowa City, IA) for the gifts of molecular constructs used in this study.

Table S2. **Mass spectrometry result of TAP tagged-VPS41 pulldown**

Gene symbol	Total peptides	Total unique peptide number
VPS41	529	67
VPS18	117	43
VPS16	107	42
VPS11	60	40
VPS33A	93	39
HNRNPU	41	29
HSPA5	32	27
TGFBRAP1	51	26
IQGAP1	24	22
PRKDC	22	22
HSPA8	45	21
CAD	21	21
HNRNPM	30	20
IRS4	27	19
LMNA	20	19
HSPA1A	27	18
RPS3	27	18
HNRNPA2B1	22	17
MATR3	20	17
RPS4X	21	16
CCT2	19	16
ATAD3A	18	16
CALM1	128	15
TUBB2A	29	15
GIGYF2	17	15
PRRC2A	16	15
RBM14	16	15
TUBA1A	33	14
RPS3A	20	14
TCP1	15	14
DHX9	15	14
FASN	15	14
CCT8	14	14
EEF1A1	19	13
CCT3	16	13
CCT7	15	13
DDX5	13	13
IGF2BP1	15	12
ILF3	13	12
RPS19	13	12
DDX17	12	12
HSPA1L	25	11
MCM7	14	11
HSPD1	11	10
DDX3Y	10	10
HSPA9	11	9
TNRC6B	11	9
FAM120A	11	9
PRRC2B	10	9
RBMX	10	9
CCT5	9	9
CDC5L	9	9
ATP5A1	9	9
HNRNPH1	13	8
RPS13	9	8
SF3B2	9	8
YLPM1	8	8
TMPO	8	8
PHGDH	8	8
MAGED2	8	8
RPL7A	8	8

Table S2. **Mass spectrometry result of TAP tagged-VPS41 pulldown** *(Continued)*

Gene symbol	Total peptides	Total unique peptide number
LRPPRC	8	8
HSPA2	10	7
HNRNPA1L2	10	7
HNRNPH3	10	7
KHSRP	9	7
ACTA2	9	7
ILF2	8	7
TUFM	8	7
PFKP	8	7
RPS7	8	7
RPS18	8	7
PIKFYVE	8	7
HUWE1	8	7
DDX21	7	7
RPL31	7	7
PSMD3	7	7
DHX15	7	7
PRPF4	7	7
RUVBL1	7	7
ELAVL1	7	7
RPL4	7	7
ACACA	7	7
RPS11	7	7
HIST1H1C	13	6
YBX1	10	6
HIST1H4A	10	6
TRIM28	8	6
RAVER1	7	6
PFKL	7	6
HIST1H1B	7	6
XRN2	7	6
RPS14	7	6
RPL7	7	6
PRRC2C	7	6
SLC25A4	7	6
EXOSC10	6	6
ATP1A1	6	6
HNRNPA3	6	6
POLDIP3	6	6
ATP5C1	6	6
HADHA	6	6
CCT6A	6	6
RPS2	6	6
RPS6	6	6
PRPF3	6	6
COPA	6	6
UBA52	14	5
TUBB	8	5
RAVER1	8	5
TUBB1	8	5
SF1	8	5
DNAJA1	7	5
RPL3	7	5
STOML2	6	5
CCT4	6	5
RPL23A	6	5
PUF60	5	5
SLC25A13	5	5
ZNF326	5	5
ANXA2	5	5
RPLP0P6	5	5

Table S2. **Mass spectrometry result of TAP tagged-VPS41 pulldown** *(Continued)*

Gene symbol	Total peptides	Total unique peptide number
KPNB1	5	5
NUMB	5	5
RPS8	5	5
VIM	5	5
RPS16	5	5
AMOT	5	5
DDX27	5	5
DDX3X	5	5
RTCB	5	5
IK	5	5
RPL8	5	5
HNRNPC	9	4
RPL23	8	4
SLC25A5	6	4
HNRNPA1	5	4
DNAJA2	5	4
H1F0	5	4
RPS15A	5	4
RPL11	5	4
HSP90AB2P	5	4
CAND1	5	4
RAN	5	4
ZC3HAV1	5	4
SUGP2	5	4
RPL5	5	4
ATP2B1	4	4
MRPL11	4	4
FAR1	4	4
RANBP2	4	4
HMGB1	4	4
NPM1	4	4
ATAD3B	4	4
LARP4B	4	4
TROVE2	4	4
NOP58	4	4
SLC25A3	4	4
NCOA3	4	4
U2SURP	4	4
STAU1	4	4
PRPF19	4	4
PUM1	4	4
HSP90AB1	4	4
RUVBL2	4	4
STX17	4	4
PCNA	4	4
RPS25	4	4
RPL26L1	4	4
RPS5	4	4
HMGB1P1	4	4
DYNC1H1	4	4
HIST3H3	4	4
CALM2	12	3
TUBB4A	7	3
HIST1H1A	6	3
MYBBP1A	5	3
ACTB	5	3
MRPS27	4	3
HSP90AA1	4	3
RPS24	4	3
EIF4G2	4	3
TUBB6	4	3

Table S2. **Mass spectrometry result of TAP tagged-VPS41 pulldown** *(Continued)*

Gene symbol	Total peptides	Total unique peptide number
TFG	4	3
RPL13	4	3
ATP2A2	4	3
LMNB1	4	3
RPL21	4	3
RPL38	4	3
HIST1H2BA	4	3
UBR5	3	3
HNRNPF	3	3
COPB1	3	3
RPL12	3	3
FUS	3	3
HNRNPR	3	3
EEF2	3	3
SLC3A2	3	3
RPS20	3	3
EIF4E2	3	3
RPLP0	3	3
EMD	3	3
HELZ	3	3
EIF4ENIF1	3	3
AZGP1	3	3
KIF5A	3	3
RPS10P5	3	3
FIP1L1	3	3
ATP5B	3	3
NUP88	3	3
DIS3	3	3
HBA1	3	3
RBM39	3	3
PPP2R1A	3	3
DPM1	3	3
MRPS18B	3	3
PPP1R13L	3	3
SAMHD1	3	3
NTPCR	3	3
HNRNPCL3	3	3
CKAP5	3	3
NONO	3	3
HNRNPUL2	3	3
RPL9	3	3
AP2M1	3	3
COPG1	3	3
PFKM	3	3
RPS27	3	3
NUP153	3	3
RPS9	3	3
RPL26	3	3
RPL35	3	3
NUP214	3	3
RPN1	3	3
RPL27	3	3
GTPBP4	3	3
CAPN1	3	3
RPL6	3	3
MYLK2	16	2
TUBB3	9	2
RPL37A	4	2
U2AF2	3	2
NXF1	3	2
RPL24	3	2

Table S2. **Mass spectrometry result of TAP tagged-VPS41 pulldown** *(Continued)*

Gene symbol	Total peptides	Total unique peptide number
HIST1H2BB	3	2
CHTOP	3	2
RPL10	3	2
HIST1H2AA	3	2
HNRNPA0	3	2
BCAS2	3	2
C17orf85	3	2
HNRNPDL	3	2
CAPRIN1	3	2
HELLS	3	2
CALML3	3	2
TIMM50	2	2
RPL19	2	2
RPS17L	2	2
RPLP2	2	2
SPTLC1	2	2
RPL18	2	2
RFC4	2	2
NSUN2	2	2
HNRNPK	2	2
GNL3L	2	2
ABCE1	2	2
MYO1C	2	2
COPB2	2	2
PC	2	2
TBC1D4	2	2
DDX56	2	2
CKAP4	2	2
ADAR	2	2
HSPB1	2	2
HIST1H2AB	2	2
PTCD3	2	2
NCOA5	2	2
SNAP47	2	2
RPL14	2	2
GCN1L1	2	2
UBAP2L	2	2
ZFR	2	2
SMC2	2	2
DYNC1LI1	2	2
RPL27A	2	2
RPS10	2	2
PIP	2	2
YME1L1	2	2
GNL3	2	2
ATP2A1	2	2
MARS	2	2
YBX3	2	2
MCCC2	2	2
DDX18	2	2
HAX1	2	2
GTF2I	2	2
SNRPD2	2	2
AKAP12	2	2
UNC45A	2	2
ZC3H11A	2	2
GAPDH	2	2
ACSL3	2	2
SF3B1	2	2
NCL	2	2
DNAJB6	2	2

Table S2. **Mass spectrometry result of TAP tagged-VPS41 pulldown** *(Continued)*

Gene symbol	Total peptides	Total unique peptide number
SUPT16H	2	2
SART1	2	2
DDX47	2	2
DAZAP1	2	2
HBB	2	2
IRAK1	2	2
HNRNPD	2	2
MRPS23	2	2
NUP98	2	2
COL14A1	2	2
RPL15	2	2
NAT10	2	2
AIFM1	2	2
SFPQ	2	2
VAC14	2	2
IGHG1	2	2
RFC3	2	2
HSP90AB3P	2	2
SYNCRIP	2	2
SAFB	2	2
VAT1	2	2
DDB1	2	2
OSBPL9	2	2
PTPLAD1	2	2
PSMD11	2	2
SNW1	2	2
MTHFD1	2	2
CDSN	2	2
PSMA1	2	2
KLC2	2	2
RBM17	2	2
PPP3CA	2	2
NKRF	2	2
ALDH18A1	2	2
PKM	2	2
DDX50	2	2
NUDT21	2	2
SNRPB	2	2
MTPAP	2	2
DDX1	2	2
BAG2	2	2
DDX49	2	2
RPL17	2	2
HAT1	2	2
RBBP4	2	2
CAMK2D	2	2
MCM3	2	2
TECPR2	2	2
PCBP2	2	2
RFC5	2	2
CPNE1	2	2
RPL35A	2	2
MAP7D3	2	2
AMBRA1	2	2
RPL13AP3	2	2
CCT6B	2	2
XRN1	2	2
PSMA6	2	2
TXN	2	2
DIMT1	2	2

References

Garg, S., M. Sharma, C. Ung, A. Tuli, D.C. Barral, D.L. Hava, N. Veerapen, G.S. Besra, N. Hacohen, and M.B. Brenner. 2011. Lysosomal trafficking, antigen presentation, and microbial killing are controlled by the Arf-like GTPase Arl8b. *Immunity*. 35:182–193. http://dx.doi.org/10.1016/j.immuni.2011.06.009

Khatter, D., V.B. Raina, D. Dwivedi, A. Sindhwani, S. Bahl, and M. Sharma. 2015. The small GTPase Arl8b regulates assembly of the mammalian HOPS complex on lysosomes. *J. Cell Sci.* 128:1746–1761. http://dx.doi.org/10.1242/jcs.162651
